# HLA-DP^84Gly^ constitutively presents endogenous peptides generated by the class I antigen processing pathway

**DOI:** 10.1038/ncomms15244

**Published:** 2017-05-10

**Authors:** Yuki Yamashita, Mark Anczurowski, Munehide Nakatsugawa, Makito Tanaka, Yuki Kagoya, Ankit Sinha, Kenji Chamoto, Toshiki Ochi, Tingxi Guo, Kayoko Saso, Marcus O. Butler, Mark D. Minden, Thomas Kislinger, Naoto Hirano

**Affiliations:** 1 Tumor Immunotherapy Program, Campbell Family Institute for Breast Cancer Research, Campbell Family Cancer Research Institute, Princess Margaret Cancer Centre, University Health Network, Toronto, Ontario, Canada M5G 2M9; 2 Department of Immunology, University of Toronto, Toronto, Ontario, Canada M5S 1A8; 3 Department of Medical Oncology, Dana-Farber Cancer Institute, Boston, Massachusetts 02215, USA; 4 Campbell Family Cancer Research Institute, Princess Margaret Cancer Centre, University Health Network, Toronto, Ontario, Canada M5G 1L7; 5 Department of Medical Biophysics, University of Toronto, Toronto, Ontario, Canada M5G 2M9; 6 Department of Medicine, University of Toronto, Toronto, Ontario, Canada M5S 1A8; 7 Campbell Family Cancer Research Institute, Princess Margaret Cancer Centre, University Health Network, Toronto, Ontario, Canada M5G 2M9; 8 University of Toronto, Toronto, Ontario, Canada M5G 2M9

## Abstract

Classical antigen processing leads to the presentation of antigenic peptides derived from endogenous and exogenous sources for MHC class I and class II molecules, respectively. Here we show that, unlike other class II molecules, prevalent HLA-DP molecules with β-chains encoding Gly84 (DP^84Gly^) constitutively present endogenous peptides. DP^84Gly^ does not bind invariant chain (Ii) via the class II-associated invariant chain peptide (CLIP) region, nor does it present CLIP. However, Ii does facilitate the transport of DP^84Gly^ from the endoplasmic reticulum (ER) to the endosomal/lysosomal pathway by transiently binding DP^84Gly^ via a non-CLIP region(s) in a pH-sensitive manner. Accordingly, like class I, DP^84Gly^ constitutively presents endogenous peptides processed by the proteasome and transported to the ER by the transporter associated with antigen processing (TAP). Therefore, DP^84Gly^, found only in common chimpanzees and humans, uniquely uses both class I and II antigen-processing pathways to present peptides derived from intracellular and extracellular sources.

## Abstract (web summary)

MHC class I and II molecules generally present endogenous and exogenous peptides, respectively, through distinct mechanisms. Here, the authors show that the class II molecule HLA-DP^84Gly^ uses both class I and II mechanisms to constitutively present peptides.

The interactions between major histocompatibility complex (MHC)/antigen complexes on antigen-presenting cells (APCs) and T-cell receptors (TCRs) on lymphocytes are required for the initiation and maintenance of T-cell-mediated adaptive immune responses. In the classical understanding of immunology, distinct antigen processing and presentation mechanisms for MHC class I and II molecules lead to the presentation of antigenic peptides derived from different sources^1^. Intracellular proteins are processed by the proteasome into peptides, which are then transported by the transporter-associated with antigen processing (TAP) into the endoplasmic reticulum (ER), where they associate with MHC class I molecules^2^. By contrast, newly synthesized MHC class II-αβ heterodimers associate rapidly with a trimeric form of invariant chain (Ii) in the ER to form a nonamer^3^,^4^. This interaction prevents the premature loading of endogenous peptides on the MHC class II cleft and, by masking the ER retention motif of Ii, facilitates the transport of class II molecules from the ER, through the Golgi, and into endosomal compartments^5^. In the endosomes, Ii is proteolytically cleaved by endopeptidases including cathepsins, leaving a short peptide called class II-associated Ii peptide (CLIP), which continues to block the binding of peptides to the class II cleft^6^. CLIP is finally released by the action of H2-DM in mice or human leukocyte antigen-DM (HLA-DM) in humans, which later catalyses the binding of high-affinity peptides found in endosomal compartments^7^. These stabilized peptide/class II complexes are then exported to the cell surface for presentation to CD4^+^ T cells.

Both HLA class I and class II genes are highly polymorphic. Molecular and genetic studies of HLA class II molecules have implicated amino-acid polymorphisms, especially those located at the class II peptide-binding cleft, with susceptibility to various diseases. These polymorphisms most likely act by directly affecting class II–TCR or class II–peptide interactions and have been identified in multiple class II alleles^8^,^9^. DRB1 residues 71 and 74, which form the DR peptide-binding cleft, appear to be a hotspot for autoimmune disease susceptibility, including rheumatoid arthritis^10^, autoimmune thyroiditis^11^ and multiple sclerosis^12^. It was also found that polymorphisms at DQB1 residues 56 and 57, located in the DQ peptide-binding cleft, strongly contribute to both susceptibility and resistance to insulin-dependent diabetes mellitus^13^. For DP molecules, it has been reported that the polymorphic amino acid at position 84 of DPB1, which forms the DP peptide-binding cleft, is linked with susceptibility to chronic beryllium disease^14^, granulomatosis with polyangiitis (Wegener's)^15^ and acute lymphoblastic leukaemia^16^. Thus, HLA class II polymorphisms can regulate T-cell repertoire diversity, which is associated with diseases such as autoimmunity and cancer.

HLA-DP alleles can be classified into two groups, encoding either Asp (DP^84Asp^), such as in DP5 and DP8, or Gly (DP^84Gly^), such as in DP2 and DP4, at position 84 of the β-chain. In this study, we show that DP^84Gly^ molecules cannot bind Ii through the CLIP region, form multimers with Ii, nor present CLIP. Interestingly, Ii still transiently binds DP^84Gly^ molecules via a non-CLIP region(s) in a pH-sensitive manner and facilitates the transport of DP^84Gly^ from the ER to the endosomes and the endocytic pathway. Surprisingly, class II is still capable of reaching the endocytic pathway in the absence of Ii. Accordingly, DP^84Gly^ constitutively presents peptides derived from both cytoplasmic and endosome-targeted proteins regardless of the expression of Ii. Importantly, these unique antigen presentation mechanisms enable DP^84Gly^ to constitutively present intracellular peptides generated by the proteasome and transported to the ER by TAP, much like class I molecules. Thus, unlike other class II molecules, DP^84Gly^, which is only found in common chimpanzees as well as archaic and modern humans, uniquely exploits both class I and II antigen pathways to present both intracellular and extracellular peptides.

## Results

### HLA-DP whose β-chain encode Gly^84^ does not present CLIP

**Figure 1:**
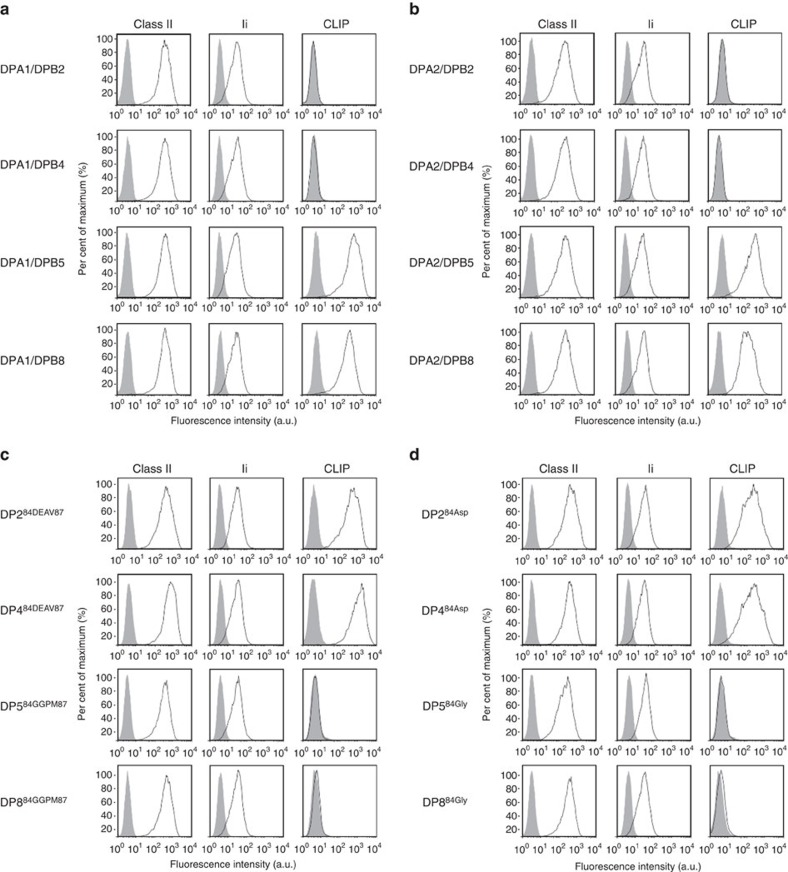
HLA-DP molecules whose β-chains encode Gly^84^ are unable to present CLIP. Surface class II, Ii and CLIP expression on T2 transfectants were analysed by flow cytometry following staining with specific monoclonal antibodies (mAbs). Note that T2 cells constitutively express Ii. (**a**,**b**) T2 cells were stably transduced with DPA1*01:03 (DPA1; **a**) or DPA1*02:01 (DPA2; **b**) in conjunction with DPB1*02:01(DPB2), DPB1*04:01 (DPB4), DPB1*05:01 (DPB5) or DPB1*08:01 (DPB8). (**c**) T2 cells were stably transfected with DPA1 along with mutated DPB2^84DEAV87^ (DP2^84DEAV87^), DPB4^84DEAV87^ (DP4^84DEAV87^), DPB5^84GGPM87^ (DP5^84GGPM87^) or DPB8^84GGPM87^ (DP8^84GGPM87^), whose amino-acid residues at positions 84–87 of the DPβ chain were substituted as indicated. (**d**) T2 cells were stably transfected with DPA1 in conjunction with mutant DPB2^84Asp^ (DP2^84Asp^), DPB4^84Asp^ (DP4^84Asp^), DPB5^84Gly^ (DP5^84Gly^) or DPB8^84Gly^ (DP8^84Gly^), where point mutations of the amino-acid residue at position 84 of each DPβ chain were substituted as indicated.

T2 cells, which endogenously express Ii but lack the expression of HLA-class II and DM, have been intensively used to study antigen processing/presentation by HLA-class II (refs 17, 18, 19, 20). To investigate HLA-DP antigen presentation mechanisms, we generated a series of DR- and DP-expressing T2 transfectants. All DR-expressing T2 cells similarly produced and presented CLIP regardless of the DR allele tested, as previously reported (Supplementary Fig. 1a)^21^. T2 cells transduced with DP5 or DP8 also presented CLIP. Surprisingly, however, cells expressing DP2 or DP4 did not present CLIP (Fig. 1a). To generate DP heterodimers, the appropriate DPβ chain was transfected along with DPA1*01:03 (DPA1) in all cases. Importantly, use of DPA1*02:01 (DPA2) in lieu of DPA1 did not affect results (Fig. 1b). In the interest of demonstrating the reproducibility of these results, additional cell lines were examined. K562 cells lack the expression of HLA-class II, Ii and HLA-DM^20^,^21^,^22^. Similar findings were obtained using a series of K562-derived transfectants expressing DR or DP alleles in conjunction with Ii (Supplementary Fig. 1b). These results suggest that lack of CLIP presentation by DP2 and DP4 is attributable to only the β-chain, but not to the α-chain nor the backbone cell utilized.

At amino-acid positions 84–87 of their DPβ chain, which constitute the open end of the DP peptide cleft, DP2 and DP4 encode GGPM (DP^84GGPM87^), while DP5 and DP8 harbour DEAV (DP^84DEAV87^)^23^,^24^. We generated T2 transfectants expressing the mutated DP alleles of DP2^84DEAV87^, DP4^84DEAV87^, DP5^84GGPM87^ or DP8^84GGPM87^, whose amino-acid residues at positions 84–87 of the β-chain were exchanged as indicated. While the ability to present CLIP was gained by T2/DP2^84DEAV87^ and T2/DP4^84DEAV87^, it was lost by T2/DP5^84GGPM87^ and T2/DP8^84GGPM87^ (Fig. 1c). These results suggest that the ^84^DEAV^87^ sequence of the DPβ chain is required and sufficient for the presentation of CLIP by HLA-DP. Point mutational analysis of DPβ chains at positions 84–87 revealed that Asp^84^ alone is necessary and sufficient for HLA-DP to present CLIP (Fig. 1d). Point mutations at residues 85–87 did not affect CLIP presentation. Since the four polymorphic positions at 84–87 of the DPβ chain are in 100% linkage disequilibrium^24^, we utilized DPβ alleles with these four amino-acid substitutions as mutant HLA-DP alleles throughout the study.

### HLA-DP^84GGPM87^ does not produce CLIP

**Figure 2:**
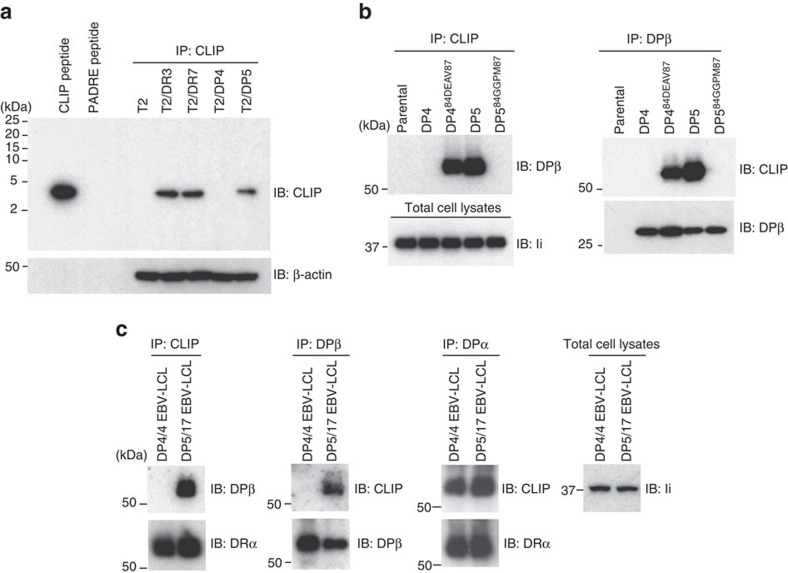
CLIP is produced by DR and DP5 but not DP4. (**a**–**c**) Total cell lysates were immunoprecipitated and immunoblotted with indicated mAbs. Total cell lysates were prepared from T2 transfectants expressing the indicated class II as a single class II allele (**a**,**b**) and EBV-LCL homozygous for DP^84DEAV87^ (DP5/17) or DP^84GGPM87^ (DP4/4) (**c**).

A lack of CLIP production by DP^84GGPM87^ could explain the observed absence of CLIP presentation on the cell surface. To investigate, anti-CLIP immunoprecipitates from lysates of T2 transfectants expressing DR3, DR7, DP4 or DP5 as a single HLA-class II allele were immunoblotted with anti-CLIP monoclonal antibody. CLIP was detectable in DR- and DP5- but not DP4-expressing T2 cells (Fig. 2a). Accordingly, DP/CLIP complexes were not formed in T2 cells expressing DP^84GGPM87^-type molecules such as T2/DP4 and T2/DP5^84GGPM87^ when anti-CLIP immunoprecipitates were immunoblotted with anti-DPβ monoclonal antibody, while those cells expressing DP^84DEAV87^ such as T2/DP5 and T2/DP4^84DEAV87^ did produce such complexes (Fig. 2b). We then performed similar experiments using Epstein-Barr virus-transformed lymphoblastoid cells (EBV-LCL), which endogenously express DR and DQ in addition to DP, to assess whether DP4 can present CLIP produced in the presence of other class II alleles in a *trans*-manner (Fig. 2c). Consistent with prior results, while DR/CLIP complexes were generated in EBV-LCL homozygous for both DP^84GGPM87^ (DP4/4) and DP^84DEAV87^ (DP5/17), DP/CLIP complexes were detected in only DP5/17, but not in DP4/4, EBV-LCL.

The above immunochemical analyses may not have sufficient sensitivity to detect low amounts of CLIP produced within cells. To directly confirm that CLIP is not produced by DP4, DP molecules were purified from T2/DP4 and T2/DP5 cells, and DP-bound peptides were acid-eluted and then subjected to mass spectrometry analysis. Again, CLIP was identified in DP5-positive, but not DP4-positive, T2 cells (Table 1). We also analysed DP-bound peptides in DP4/4 and DP5/17 EBV-LCL through mass spectrometry analysis. CLIP was detected in DP5/17 but not in DP4/4 EBV-LCL (Supplementary Table 1). Note that because of HLA-DM expression the intensity of CLIP found in DP5/17 EBV-LCL is substantially lower than in T2/DP5 cells. These results together demonstrate that DP^84GGPM87^ such as DP4 cannot produce or present CLIP on its own, nor present CLIP produced by other class II.

### DP^84GGPM87^ cannot form a nonamer with Ii within cells

**Figure 3:**
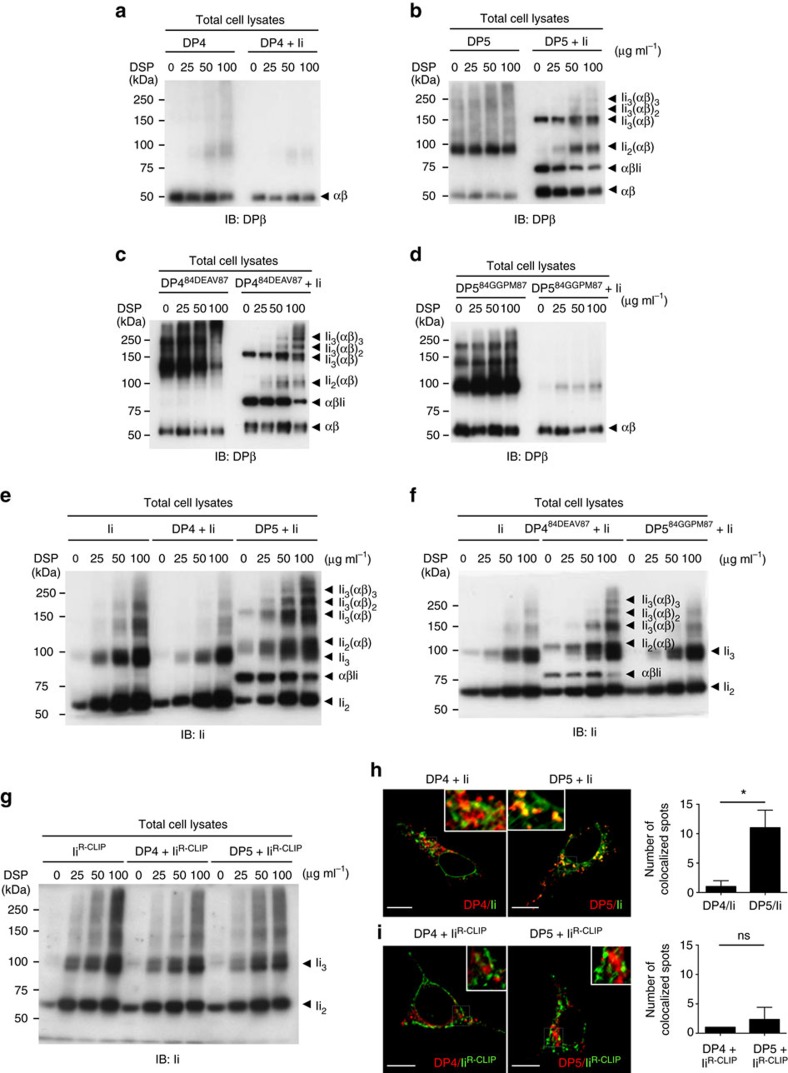
DP^84DEAV87^ but not DP^84GGPM87^ molecules form a nonamer complex with Ii via the CLIP region. (**a**–**g**) HEK293 cells were transiently transfected with the indicated combinations of genes. Cells were treated with DSP, a chemical crosslinker, at the indicated concentrations for 2 h. Non-reduced samples were immunoblotted with anti-DPβ (**a**–**d**) or Ii (**e**–**g**) mAb. (**h**,**i**) HEK293 cells were transfected with the indicated combinations of genes. Fixed cells were permeabilized and stained for Ii (**h**) or Ii^R-CLIP^ (**i**; green) and DP (red), and then analysed by confocal microscopy. Inset boxes indicate the areas shown at higher magnification. Note that HEK293 cells are deficient in class II and Ii expression. Scale bar in all images, 10 μm. Quantification of co-localized spots represents means±s.d. of three counted cells in each condition. ns, not significant; *P<0.05 by unpaired, two-tailed Welch's *t*-test.

Typically, three class II αβ-heterodimers associate with a trimer of Ii to generate a nonamer in the ER^3^,^4^. This nonameric complex is dependent on CLIP-mediated interaction between Ii and class II, and is a precursor to CLIP production and presentation on class II (ref. 25). To investigate whether DP^84GGPM87^ forms such a nonamer and can interact with Ii through the CLIP region, HEK293 cells, deficient for class II, Ii and HLA-DM, were transiently transfected with combinations of DP and Ii as previously reported^26^,^27^. Following chemical crosslinking, lysates were immunoblotted with anti-DPβ monoclonal antibody (Fig. 3a–d). DP^84DEAV87^-type alleles, that is, DP5 (Fig. 3b) and DP4^84DEAV87^ (Fig. 3c), formed several intermediate multimers with Ii such as Ii_3_(αβ)_2_, Ii_3_(αβ), Ii_2_(αβ) and αβIi, in addition to the prototypic nonamer Ii_3_(αβ)_3_, with increasing dithiobis succinimidyl propionate (DSP) concentration. In contrast, no complex formation was observed between Ii and DP^84GGPM87^-type class II, that is, DP4 (Fig. 3a) or DP5^84GGPM87^ (Fig. 3d). Similar results were obtained when blotting with anti-Ii monoclonal antibody (Fig. 3e,f) and using T2 transfectants, in which DP^84DEAV87^, but not DP^84GGPM87^, formed a nonamer with endogenous Ii (Supplementary Fig. 2). These results demonstrate that, unlike DP^84DEAV87^ or DR, DP^84GGPM87^ cannot form a nonamer with Ii and that the ^84^DEAV^87^ region of the β-chain is required for DP/Ii nonamer formation. Confirming the necessity of the CLIP region in this interaction, neither DP4 nor DP5 generated a nonamer with Ii^R-CLIP^, a mutant Ii chain carrying a reversed CLIP region sequence (Fig. 3g). In addition, we confirmed that DP5, but not DP4, could co-localize with Ii through confocal microscopy analysis (Fig. 3h), but that neither DP4 nor DP5 could co-localize with Ii^R-CLIP^ (Fig. 3i). These results demonstrate that the lack of CLIP production and presentation to the cell surface is derived from the inability of Ii to associate with DP^84GGPM87^ through its CLIP region. In addition, this interaction with the CLIP region is required for both nonamer formation and the colocalization of Ii with DP.

### Ii can still facilitate the egress of DP^84GGPM87^ from the ER

**Figure 4:**
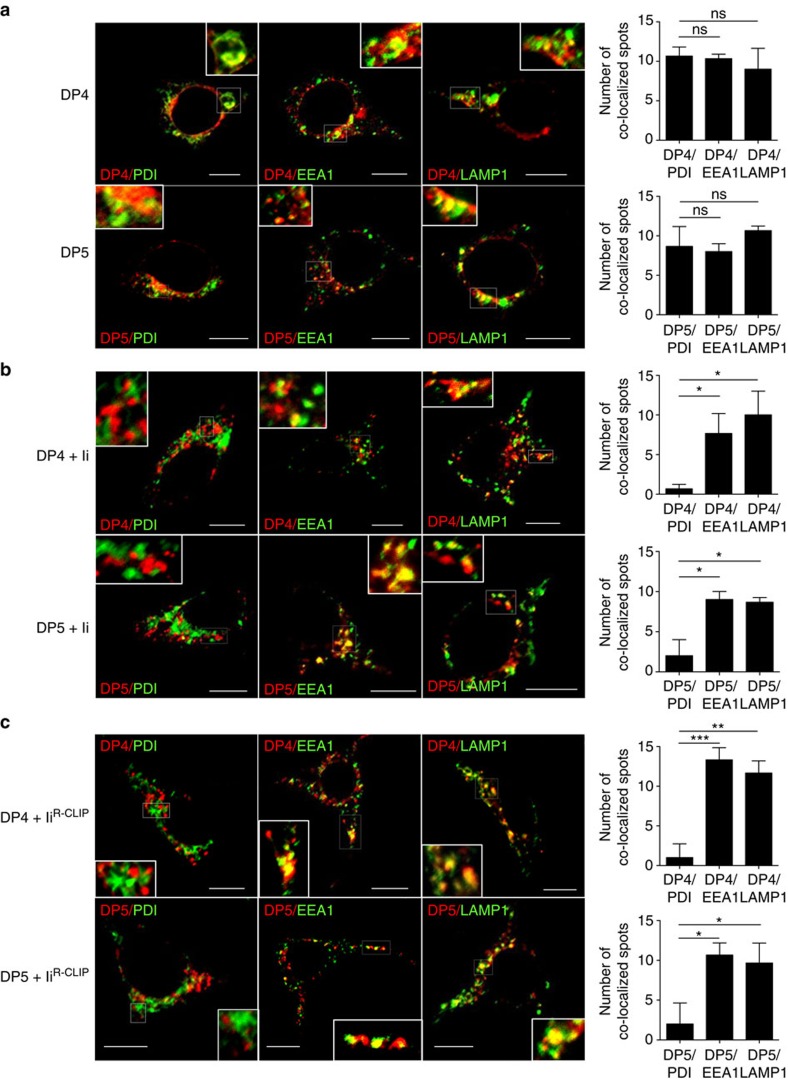
Subcellular localization of DP4 and DP5 in the presence or absence of Ii. (**a**–**c**) HEK293 cells were transfected with the indicated combinations of genes. Fixed cells were permeabilized and stained for DP (red) in conjunction with an ER marker, PDI (green, left panels), an early endosomal marker, EEA1 (green, middle panels) or a late endosomal/lysosomal marker, LAMP-1 (green, right panels) and analysed by confocal microscopy. Inset boxes indicate the areas depicted at higher magnification. Scale bar in all images, 10 μm. Quantification of co-localized spots represents means±s.d. of three counted cells in each condition. ns, not significant; *P<0.05, **P<0.01, ***P<0.001 by unpaired, two-tailed Welch's *t*-test.

The formation of class II/Ii complexes is important for class II's trafficking to the endocytic pathway and/or cell surface^5^. To study a potential role for Ii in the egress of DP^84GGPM87^ from the ER, the subcellular localization of both DP4 and DP5 in the presence or absence of Ii was analysed in HEK293 cells by confocal microscopy (Fig. 4). The expressions of class II, Ii and CLIP on HEK293 transfectants were confirmed by flow cytometry analysis (Supplementary Fig. 3). Both DP4 and DP5 were similarly distributed in the ER, early endosomes and lysosomes in the absence of Ii (Fig. 4a). Surprisingly, despite the lack of CLIP-mediated binding between DP4 and Ii, the forced co-expression of Ii similarly facilitated the transport of both DP4 and DP5 from the ER to the early endosomes/lysosomes, as neither DP4 nor DP5 localized in the ER in the presence of Ii (Fig. 4b). These results demonstrate that irrespective of the sequences at positions 84–87, Ii promotes the transport of DP from the ER to the early endosomes/lysosomes. Furthermore, these results suggest that the CLIP region is not required for Ii to facilitate the egress of DP from the ER, since DP4 cannot make a nonamer with Ii via the CLIP region as described above. To confirm this, HEK293 cells were transfected with Ii^R-CLIP^ along with either DP4 or DP5. Once again, both DP4 and DP5 localized in the early endosomes and lysosomes but not in the ER (Fig. 4c). These results demonstrate that Ii can facilitate the egress of both DP^84GGPM87^ and DP^84DEAV87^ in a non-CLIP region-dependent manner, without forming a nonamer through the CLIP region.

### DP^84GGPM87^ associates with Ii in a pH-dependent manner

**Figure 5:**
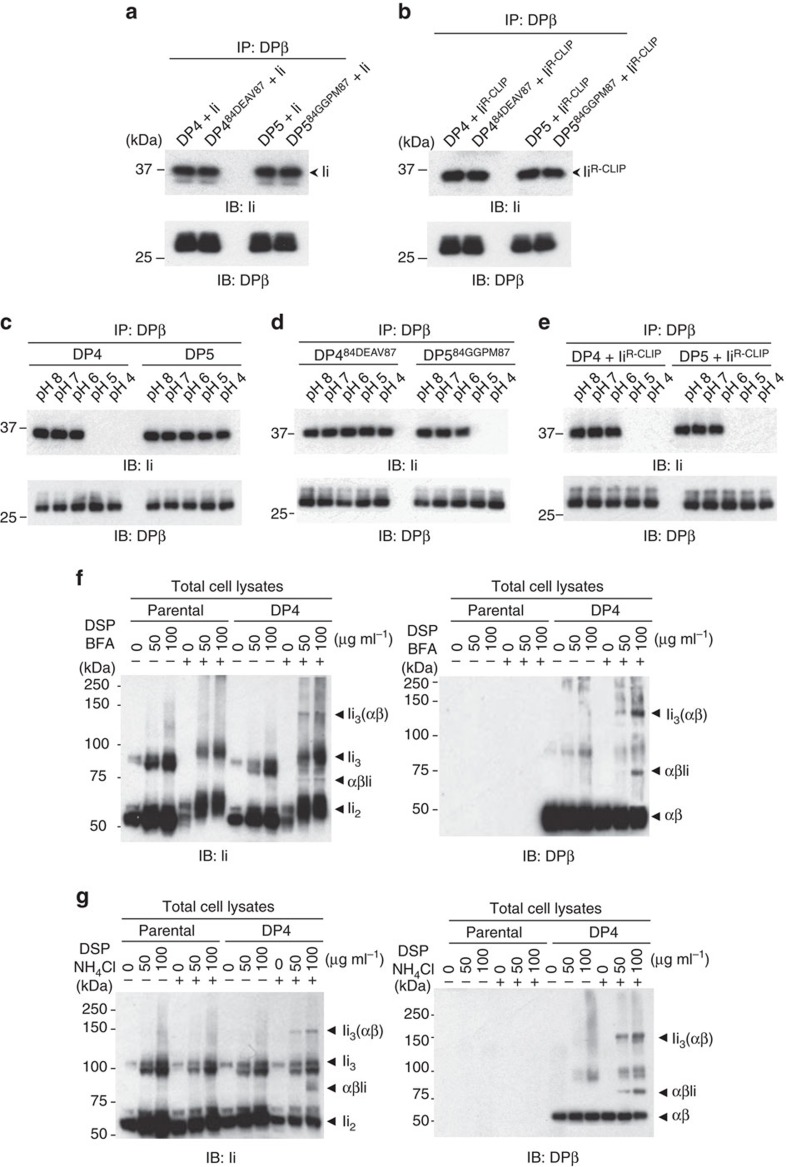
DP^84GGPM87^ binds to Ii via a non-CLIP region(s) in neutral pH conditions. (**a**,**b**) HEK293 cells were transfected with the indicated combinations of genes. Total cell lysates were immunoprecipitated with anti-DPβ mAb and immunoblotted with anti-Ii or DPβ mAb. (**c**–**e**) Lysates of T2/DP transfectants (**c**,**d**) or HEK293 cells transfected with the indicated combinations of DP and Ii^R-CLIP^ genes (**e**) were immunoprecipitated with anti-DPβ mAb. Note that T2 cells naturally express Ii. The immunoprecipitates were washed with buffer of graded pH as indicated and immunoblotted with anti-Ii or DPβ mAb. (**f**,**g**) T2 and T2/DP4 were cultured in the presence or absence of 10 μg ml^−1^ BFA (**f**) or 40 mM NH_4_Cl (**g**). Cells were further treated with DSP at the indicated concentrations for 2 h. Non-reduced samples were immunoblotted with anti-Ii (left) or DPβ (right) mAb.

The observation that Ii can still facilitate the egress of DP^84GGPM87^-type class II from the ER despite the lack of CLIP–region association led to the question of how the nature of the DP/Ii association differs between DP^84GGPM87^ and DP^84DEAV87^-type class II molecules. In line with Ii's ability to facilitate the egress of both DP^84DEAV87^ and DP^84GGPM87^ from the ER to the endocytic pathway, anti-Ii immunoblotting of DPβ immunoprecipitates confirmed that both DP^84DEAV87^ and DP^84GGPM87^ can bind Ii (Fig. 5a) as well as Ii^R-CLIP^ (Fig. 5b) *in vitro*. To examine whether these associations differ under physiologically relevant conditions, we decided to examine the formation of these complexes across a range of pH levels. The secretory pathway organelles through which class II is known to travel exhibit a gradient of decreasing pH. This pH gradient begins at near neutrality in the ER (pH 7.1–7.2; ref. 28), becomes mildly acidic in the Golgi (pH 6.2–7.0; ref. 29), and reaches higher acidity within the secretory granules such as the endosomes/lysosomes (pH 5.0; ref. 30). To investigate Ii's ability to associate with DP alleles across a range of physiological pH conditions, we compared the acid sensitivity of the association between Ii and either DP^84DEAV87^ or DP^84GGPM87^. Binding between Ii and DP^84DEAV87^-type molecules, that is, DP5 and DP4^84DEAV87^, was proven to be stable in acidic conditions, even at pH 4 (Fig. 5c,d). In contrast, DP^84GGPM87^-type class II, namely DP4 and DP5^84GGPM87^, completely dissociated from Ii at pH 5. Binding of Ii^R-CLIP^ to both DP4 and DP5 via a non-CLIP region(s) was also abrogated at pH 5 (Fig. 5e). These results suggest that, while the CLIP-dependent interaction between Ii and DP^84DEAV87^ is acid-resistant, the CLIP-independent association between Ii and DP is acid-sensitive.

These results raised the possibility that DP^84GGPM87^ transiently associates with Ii in the ER at neutral pH in a CLIP-independent manner, and that upon transport from the ER to the early endosomes/lysosomes it rapidly dissociates as the pH becomes acidic. To accumulate and visualize transient and intermediate complexes that are not detectable under physiological conditions in support of this concept, T2 cells expressing DP^84GGPM87^ (DP4 or DP5^84GGPM87^) were treated with brefeldin A (BFA), which inhibits protein transport from the ER to the endosomes, and the chemical crosslinker DSP. Blotting with anti-Ii and DPβ monoclonal antibodies demonstrated that DP^84GGPM87^ formed Ii_3_(αβ) and αβIi complexes with Ii within the ER (Fig. 5f and Supplementary Fig. 4a). The same T2 transfectants were treated with ammonium chloride (NH_4_Cl), which increases pH in the endosomes, and subsequently with the chemical crosslinker DSP. Both anti-Ii and DPβ monoclonal antibodies clearly detected the formation of Ii_3_(αβ) and αβIi complexes between Ii and DP^84GGPM87^ within the endosomes with elevated pH (Fig. 5g and Supplementary Fig. 4b). Higher-order multimeric complexes including Ii_3_(αβ)_2_ and Ii_3_(αβ)_3_ were not formed by treatment with BFA or NH_4_Cl, suggesting that CLIP-dependent binding is required for the generation of these higher-order multimers. Nevertheless, these results indicate that DP^84GGPM87^ and Ii transiently form acid-sensitive complexes via a non-CLIP region(s) in the ER, and that these complexes rapidly disassemble upon their exit from the ER and entry into the early endosomes.

### DP^84GGPM87^ presents peptides made by the proteasome and TAP

**Figure 6:**
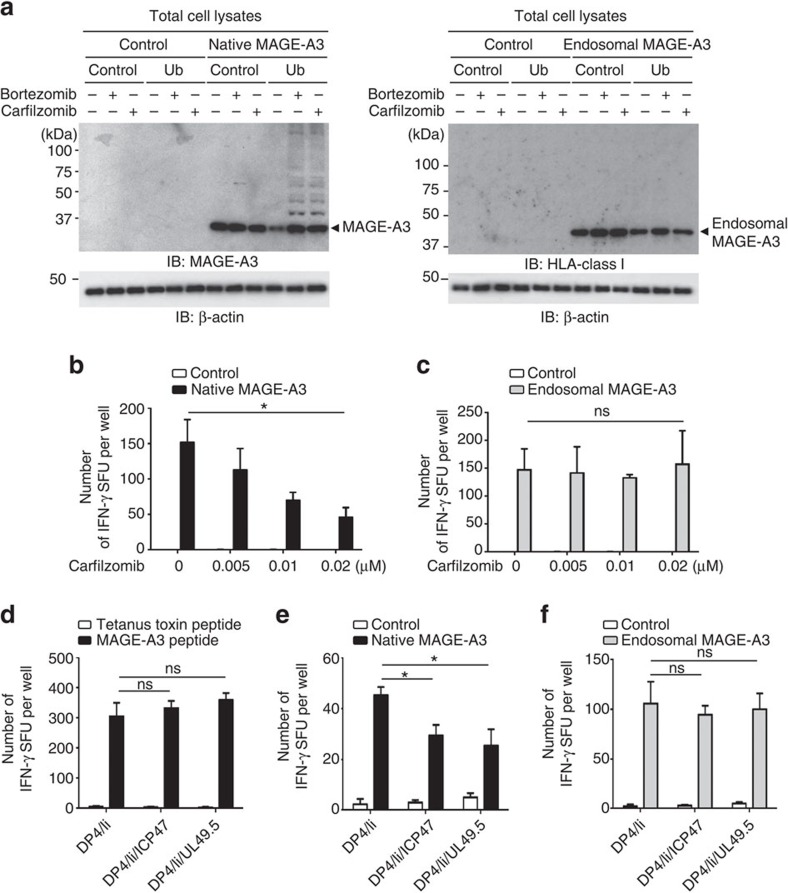
DP^84GGPM87^-expressing cells constitutively present intracellular peptides generated by the proteasome and TAP-dependent pathway. (**a**) K562 aAPCs were transiently transfected with the indicated combinations of genes and cultured in the presence or absence of 0.02 μM bortezomib or 0.02 μM carfilzomib for 48 h. Total cell lysates were immunoblotted with anti-MAGE-A3, anti-HLA-class I or anti-β-actin mAb. (**b**,**c**) K562/DP4/Ii cells were transiently transfected with a retrovirus vector encoding IRES-EGFP (control), or a native (**b**) or endosome-targeted (**c**) form of MAGE-A3 linked with IRES-EGFP. Cells were then cultured with carfilzomib at the indicated concentrations for 48 h. Transient transfection efficiencies were normalized to EGFP expression measured by flow cytometry. DP4/MAGE-A3_243–258_ CD4^+^ T cells were stimulated with the K562/DP4/Ii transfectants and IFN-γ secretion was measured by ELISPOT analysis. Data shown represent means±s.d.'s of triplicates. (**d**) DP4/MAGE-A3_243–258_ CD4^+^ T cells were stimulated with the indicated K562-based aAPCs pulsed with tetanus toxin_947–967_ (control) or MAGE-A3_243–258_ peptide, and IFN-γ secretion was evaluated by ELISPOT assays. Data shown represent means±s.d.'s of triplicates. (**e**,**f**) The indicated K562-based aAPCs were transiently transfected with a retrovirus vector encoding IRES-EGFP (control) or a native (**e**) or endosome-targeted (**f**) form of MAGE-A3 linked with IRES-EGFP. Transient transfection efficiencies were normalized to EGFP expression measured by flow cytometry. DP4/MAGE-A3_243–258_ CD4^+^ T cells were stimulated with the indicated aAPCs and IFN-γ secretion was measured by ELISPOT analysis. Data shown represent means±s.d.'s of triplicates. Results are representative of three independent experiments. ns, not significant; *P<0.05 by unpaired, two-tailed Welch's *t*-test.

MHC-class I-presented peptides are predominantly generated by the proteasome and then transported from the cytosol to the ER by TAP. On the basis of the observations above, we hypothesized that DP^84GGPM87^-type class II can uniquely present intracellular peptides produced by the processing pathway involving the proteasome and TAP in a similar manner to class I molecules, taking advantage of the lack of CLIP–region binding to these class II. To differentiate between peptides produced by the endogenous versus exogenous antigen processing and presentation pathways separately, we generated both endogenous and exogenous model antigens. We utilized cytoplasmic MAGE-A3 protein as an endogenous model antigen, whose MAGE-A3_243–258_ peptide is naturally processed and presented by DP4 (ref. 31). Mutant MAGE-A3 protein targeted to the endosomal/lysosomal pathway was used as a control exogenous model antigen. Class I- and II-defective K562 cells, used as a backbone of artificial APCs (aAPCs), are equipped with the normal proteasome machinery, and are thereby capable of endogenously processing and presenting class I-restricted peptides when transduced with a class I gene^32^. To examine whether inhibition of proteasome function prevents the production of peptides derived from ubiquitinated cytoplasmic proteins, K562 aAPCs were transfected with the native form or endosome-targeted form of the *MAGE-A3* gene along with the ubiquitin gene, and treated with a proteasome inhibitor, either carfilzomib or bortezomib. Accumulation of polyubiquitinated, non-degraded MAGE-A3 protein was observed when the native, but not endosome-targeted, form of MAGE-A3 was transfected and treated with a proteasome inhibitor (Fig. 6a). The effect of the proteasome inhibitor on antigen processing was determined by measuring DP4/MAGE-A3_243–258_ peptide-specific T-cell responses to these aAPCs by ELISPOT analysis. Dose-dependent reduction in T-cell responses by the proteasome inhibitors were seen only when K562/DP4/Ii were transfected with the native form of MAGE-A3 and used as stimulators (Fig. 6b and Supplementary Fig. 5a). In contrast, K562/DP4/Ii cells transduced with the endosome-targeted form of MAGE-A3-induced consistent T-cell responses, unaffected by proteasome inhibitor treatment (Fig. 6c and Supplementary Fig. 5b).

The herpes virus-derived genes *ICP47* and *UL49.5* are known to suppress TAP function^33^,^34^. To confirm the effect of ICP47 and UL49.5 on the transport of class I-restricted peptides within K562 cells, K562 cells expressing A2 were transduced with either ICP47 or UL49.5 (Supplementary Fig. 5c). These K562/A2 transfectants induced a similar degree of A2/MART1_27–35_-specific T-cell responses when exogenously pulsed with A2/MART1_27–35_ peptide (Supplementary Fig. 5d). However, the forced expression of ICP47 or UL49.5 reduced the natural processing and presentation of A2/MART1_27–35_ peptide derived from the full-length *MART1* gene (Supplementary Fig. 5e). These results indicate that ICP47 and UL49.5 efficiently inhibited the transport of class I-restricted peptides to the ER within K562 cells. To study whether TAP molecules were also involved in the transport of DP4-restricted peptides, K562/DP4/Ii cells were transduced with ICP47 or UL49.5 (Supplementary Fig. 5f). These K562/DP4/Ii transfectants induced a similar magnitude of specific DP4/MAGE-A3_243–258_ T-cell responses when exogenously pulsed with MAGE-A3_243–258_ peptide (Fig. 6d). However, significantly reduced DP4/MAGE-A3_243–258_ responses were observed in the presence of ICP47 or UL49.5 only when these transfectants expressed the native form of MAGE-A3 (Fig. 6e). On the other hand, ICP47 or UL49.5 did not affect the presentation of DP4/MAGE-A3_243–258_ peptide derived from the endosome-targeted form MAGE-A3 (Fig. 6f). These results demonstrate that DP4 can present intracellular peptides processed by the proteasome and transported to the ER by TAP in a similar manner to class I molecules.

### DP^84GGPM87^ presents multisource peptides regardless of Ii

**Figure 7:**
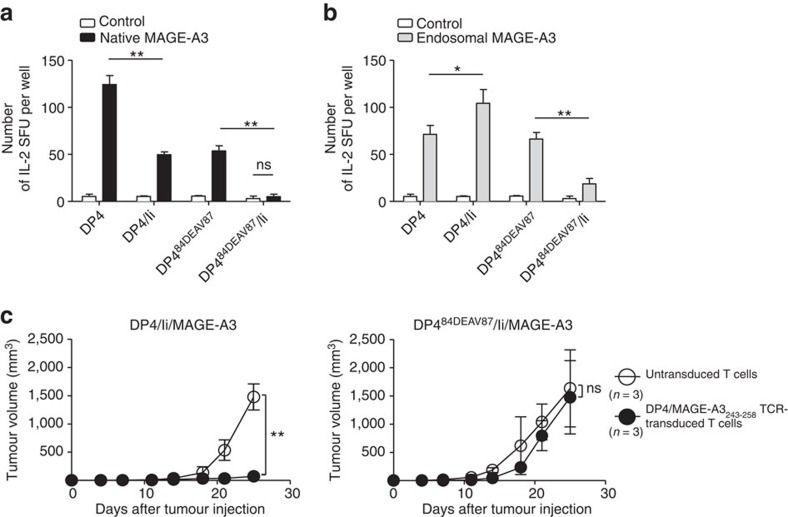
DP^84GGPM87^ but not DP^84DEAV87^ constitutively presents peptides derived from intracellular proteins regardless of Ii expression. (**a**,**b**) The indicated K562-based aAPCs were transiently transfected with a retrovirus vector encoding IRES-EGFP (control) or a native (**a**) or endosome-targeted (**b**) form of MAGE-A3 linked with IRES-EGFP. Transient transfection efficiencies were normalized to EGFP expression measured by flow cytometry. DP4/MAGE-A3_243–258_ CD4^+^ T cells were stimulated with the indicated APCs and IL-2 secretion was measured by ELISPOT analysis. Data shown represent means±s.d.'s of triplicates. (**c**) NSG mice were subcutaneously inoculated with 2 × 10^6^ K562 cells stably expressing DP4/Ii/MAGE-A3 or DP4^84DEAV87^/Ii/MAGE-A3. Two days later, the mice were treated with 4 × 10^7^ CD3^+^ T cells untransduced or transduced with DP4/MAGE-A3_243–258_ TCR. The mean tumour size for each group is represented as the average±s.d. of three mice. There was no significant difference in the tumorigenicity of the two cell lines (data not shown). Results are representative of three independent experiments. ns, not significant; *P<0.05, **P<0.01 by unpaired, two-tailed Welch's *t*-test.

To demonstrate that cells expressing DP^84GGPM87^ such as DP4 constitutively present CD4^+^ T-cell epitopes derived from both intracellular and extracellular proteins regardless of Ii expression, a series of K562-based aAPCs expressing DP4 or DP4^84DEAV87^ in the presence or absence of Ii were generated (Supplementary Fig. 6a)^20^,^21^. We then compared DP4/MAGE-A3_243–258_ peptide-specific T-cell responses against these DP4- and DP4^84DEAV87^-expressing target cells. K562/DP4 very efficiently presented MAGE-A3_243–258_ peptide derived from both native (Fig. 7a) and endosome-targeted (Fig. 7b) MAGE-A3 in both the presence and absence of Ii. Ii significantly suppressed the presentation of MAGE-A3_243–258_ peptide derived from both forms of MAGE-A3 by DP4^84DEAV87^ (Fig. 7a,b), since Ii-derived CLIP occupied the DP4^84DEAV87^ cleft and prevented its loading with MAGE-A3_243–258_ peptide (Fig. 1c). Note that, since K562 cells are deficient in the endogenous expression of HLA-DM, the CLIP peptide cannot be removed from the class II cleft and is instead presented to the cell surface^20^,^21^. The presence of Ii also reduced the presentation of MAGE-A3_243–258_ peptide derived from native MAGE-A3 by DP4 (Fig. 7a), probably because Ii facilitated the egress of DP from the ER resulting in less efficient loading of MAGE-A3_243–258_ peptide onto DP4 in the ER, where this loading is presumed to occur (Fig. 4a,b). In contrast, when endosome-targeted MAGE-A3 was expressed along with Ii, K562/DP4 was able to present MAGE-A3_243–258_ peptide more efficiently compared to when Ii was absent (Fig. 7b). This is probably because the co-expressed Ii facilitated the transport of DP4 to the endosomal/lysosomal pathway where DP4 is supposed to be loaded with MAGE-A3_243–258_ peptide derived from endosome-targeted MAGE-A3 (Fig. 4a,b). These results suggest that DP^84GGPM87^ can constitutively present CD4^+^ T-cell epitopes derived from both intracellular and extracellular proteins regardless of Ii expression, since the loading of DP^84GGPM87^ with peptides is not prevented by binding with CLIP. *In vitro* and *in vivo* cytotoxicity assays also confirmed that DP4 but not DP4^84DEAV87^ can effectively present endogenous MAGE-A3_243–358_ peptide and stimulate T cells regardless of Ii expression (Fig. 7c and Supplementary Fig. 6b,c). These results clearly demonstrate that DP^84GGPM87^ can constitutively present peptides derived from both intracellular and extracellular tumour-associated antigens and is directly recognized by antigen-specific CD4^+^ T cells in a CLIP-independent manner.

## Discussion

While the classical understanding of antigen presentation dictates that MHC class II molecules present peptides from exogenous sources, we demonstrate here that a common human polymorphism results in the ability to present endogenous peptides. We have shown that DP^84GGPM87^ molecules such as DP4 cannot bind Ii via the CLIP region, generate multimers with Ii, nor present CLIP. Cells expressing DP^84GGPM87^ molecules do not produce CLIP from endogenous full-length Ii. However, they can, in turn, constitutively present both endogenous and exogenous antigens to CD4^+^ T cells. Amazingly, the single amino acid, Gly^84^, of the DPβ chain is necessary and sufficient for this lack of CLIP production and its subsequent presentation. Interestingly, DPβ chain genes encoding Gly^84^ are not found in the genomes of subhuman primates, including bonobos (*Pan paniscus*), western gorillas (*Gorilla gorilla*), bornean orangutans (*P. pygmaeus*), crab-eating macaques (*Macaca fascicularis*), nor rhesus macaques (*M. mulatta*; https://www.ebi.ac.uk/ipd/mhc/nhp/nomenclature.html; Supplementary Table 2). The Part-DPB1*02 allele of common chimpanzees (*P. troglodytes*), however, encodes ^84^GEAV^87^, which also cannot produce CLIP. In archaic humans, Neanderthals (*Homo neanderthalensis)*, the DPβ chain gene encoding ^84^GGPM^87^ has also been found and has a similar sequence to the modern human DPB1*0401 allele^35^. Thus, class II alleles that cannot form CLIP seem to have evolved in the most recent hominid ancestor of humans and common chimpanzees (*P. troglodytes*), possibly in response to evolutionary pressures imposed by infectious diseases such as the simian immunodeficiency virus. It is possible that, to combat such diseases, the development of a class II allele such as DP^84GGPM87^, which could constitutively present intracellular pathogen-derived peptides and stimulate anti-infective CD4^+^ T cells, would have provided evolutionary advantages.

We have demonstrated that the loss of CLIP–region interaction to DP^84Gly^ changes the nature of the multimeric complex formed between DP and Ii, rendering Ii dependent solely on non-CLIP, acid-sensitive interactions with class II to guide its transport through the endocytic pathway. Interestingly though, the binding between Ii and DP through a non-CLIP region(s) was sufficient to mediate Ii's transport function; neither nonamer formation nor CLIP-mediated binding was required. In other words, Ii was able to facilitate the transport of both DP^84DEAV87^ and DP^84GGPM87^ from the ER to the endosomes. However, the mechanisms that dissociate Ii/CLIP from DP still differ between DP^84DEAV87^ and DP^84GGPM87^, while multiple cleavages of Ii by endopeptidases including cathepsins and subsequent release by HLA-DM are involved for DP^84DEAV87^, an acidic pH environment alone seemed sufficient for DP^84GGPM87^ to be dissociated from Ii, at least *in vitro*.

TAP is a member of the ATP-binding-cassette transporter family. It carries endogenous peptides 8–11 amino acids in length into the ER, where they bind to nascent MHC class I molecules^36^. Surprisingly, we found that TAP was involved in DP4 antigen processing. The length of class II-bound peptides are usually longer than those of class I-bound peptides and are between ∼10 and 30 amino acids^37^. It is known, however, that peptides longer than 8–11 amino acids (up to 40 amino acid in length) can be produced by the proteasome and transported to the ER by TAP^38^. In fact, longer peptides with a length of 9–16 residues are optimal substrates for human TAP proteins^39^. These observations support our results demonstrating that DP4^84GGPM87^ molecules present peptides processed by the proteasome and delivered by TAP to the ER.

DP4 is the most frequent HLA allele, present in up to 75% of Caucasians^40^,^41^. A variety of DP4-restricted antigenic peptides that can induce CD4^+^ T-cell responses in association with cancer and infection have been identified (http://archive.cancerimmunity.org/peptidedatabase/tumorspecific.htm)^20^,^42^,^43^,^44^. Many of these peptides are derived from endogenous proteins. The unique, direct mechanism revealed in this study for DP4 or DP^84GGPM87^-specific antigen processing may result in an enhanced presentation of endogenous peptides. Considering that HLA-DP expression levels are generally lower than HLA-DR, this potentially intensified antigen presentation may compensate and result in more effective targeting by effector T cells. It should be noted that the first HLA-class II-restricted TCR gene therapy trial targeted the HLA-DP4-restricted MAGE-A3 peptide presented in this manuscript.

If DP4 or DP^84GGPM87^-restricted epitopes from self-proteins are so efficiently processed and presented, a potential ramification would obviously be the induction of toxic autoimmune responses leading to human autoimmune diseases. Given the lower expression of HLA-DP compared to other class II alleles, additional inflammatory signals may be required and limit the incidence of disease. In a genome-wide significant association study of granulomatosis with polyangiitis (Wegener's), significant associations were identified with the *HLA-DPB1* and *HLA-DPA1* genes. Importantly, the DPB1 association was fully accounted for by the DPB1*04 allele, which encodes the DP4 β chain. This result suggests that peptide(s) presented by HLA-DP4 molecules are involved in Wegener's granulomatosis, and efforts to identify them are underway. Going forward, the question arises whether DP4 or DP^84GGPM87^ is associated with other autoimmune diseases. It is possible that the DP4 or other DP^84GGPM87^ alleles are involved in a subset of autoimmune diseases, which are accompanied by strong inflammation that can upregulate HLA class II expression on APCs.

In conclusion, we have demonstrated that common chimpanzees and humans (both archaic and modern) possess HLA-DP alleles encoding Gly^84^ in their β-chain, which cannot bind Ii via the CLIP region, make nonameric complexes with Ii, nor generate/present CLIP. Intriguingly, DP^84Gly^ transiently associates with Ii via a non-CLIP region(s) in a pH-sensitive manner, which facilitates its transport from the ER to the early endosomes/lysosomes. Thus, in addition to exogenous peptides, DP^84Gly^ such as DP4, which is one of the most prevalent HLA allele in many ethnic groups, can constitutively present endogenous peptides generated by the proteasome and transported to the ER by TAP, regardless of Ii expression, representing a novel cross-presentation pathway to CD4^+^ T cells. As seen with the strong association between HLA-DP4 and Wegener's granulomatosis, the prevalence of DP^84Gly^ in the human population may have implications for health and the susceptibility to disease.

## Methods

### Reagents

DSP and ammonium chloride (NH_4_Cl) were purchased from Sigma-Aldrich (St Louis, MO). BFA was purchased from BioLegend (San Diego, CA). Bortezomib was from Selleck Chemicals (Houston, TX). Carfilzomib was purchased from ApexBio Technology (Houston, TX).

### Animal studies

NOD *scid* interleukin (IL)-2 receptor gamma chain knockout mice (NSG) were obtained from the Jackson Laboratory (Stock Number: 005557). All mice used were male, aged 6–8 weeks. All experimental procedures were approved by the Princess Margaret Cancer Centre Animal Care Committee at the University Health Network and performed in accordance with the Canadian Council on Animal Care Guidelines.

### Cells and cDNAs

Peripheral blood mononuclear cells were obtained from healthy donors following institutional review board approval. Written informed consent was obtained from all donors who provided the samples. High-resolution HLA DNA typing was performed by the American Red Cross. EBV-LCL were produced by infecting peripheral blood mononuclear cell with EBV (B95-8 strain). K562 is a human erythroleukaemic cell line, deficient in class I, class II, Ii and DM expression. T2 cells endogenously express Ii but lack the expression of HLA-class II and DM. K562 and T2 cells were cultured in RPMI1640 supplemented with 10% fetal calf serum and gentamycin (Life Technologies, Carlsbad, CA). HEK293 cells, deficient in HLA class II, Ii, and DM expression, were maintained in DMEM containing 10% fetal calf serum and gentamycin. All cell lines were obtained from American Type Culture Collection (ATCC), Manassas. All cells were routinely checked for the presence of mycoplasma contamination using the polymerase chain reaction-based Mycoplasma Detection Kit from ATCC. K562- and T2-derived cells were retrovirally transduced with cDNAs encoding HLA-class II in conjunction with Ii, and transduced cells were isolated using magnetic beads as described previously^20^,^21^. Except where otherwise stated, HLA-DP heterodimers comprises DPA1*01:03 (DPA1) in conjunction with one of DPB1*05:01 (DPB5), DPB1*08:01 (DPB8), DPB1*02:01 (DPB2) or DPB1*04:01 (DPB4) to form DP5, DP8, DP2 and DP4, respectively. A2/MART1_27–35_ TCR (clone DMF5) genes were kindly provided by Dr Rosenberg (NIH/NCI, Bethesda, MD). TCRα- and β-chain genes specific for DP4/MAGE-A3_243–258_ were cloned based upon published sequences. cDNAs were fused with a truncated version of human nerve growth factor receptor (ΔNGFR) via an optimized intervening sequence consisting of a furin cleavage site, an SGSG spacer sequence, and an F2A sequence^45^,^46^. Human ubiquitin gene was isolated based upon published sequences. ICP47 and UL49.5 were also cloned based upon published sequences and fused with IRES-ΔNGFR. Whereas ICP47 competes for peptide binding to TAP, UL49.5 inhibits critical conformational changes at a later phase of the translocation cycle, thereby both inhibiting peptide transport^33^,^34^. Native and endosome-targeted forms of MAGE-A3 were tandemly linked with IRES-EGFP for transient expression experiments^47^. For stable expression experiments, native MAGE-A3 was fused with ΔNGFR as described above. To generate an endosome-targeted form of MAGE-A3, the 236–265 amino-acid region of MAGE-A3 was in-frame ligated downstream of the HLA-A*02:01 cDNA^48^. ΔNGFR-transduced cells were isolated using anti-NGFR monoclonal antibody. All cDNAs were cloned into the pMX vector and their sequences were verified.

### Transient transfection

HEK293 and K562 cells were transiently transfected using TransIT 293 (Mirus, Madison, WI) and Lipofectamine 2000 (Life Technologies), respectively, according to the manufacturer's instruction.

### Flow cytometry analysis

Monoclonal antibodies recognizing the following surface antigens were used: pan HLA class II (6604366, 1:500, Beckman Coulter), HLA-DP (ab21119-100, 1:100, Abcam), HLA-DR (555561, 1:500, BD Biosciences), Ii (555540, 1:500, BD Biosciences), CLIP (555981, 1:200, BD Biosciences), HLA-DM (555983, 1:250, BD Biosciences), NGFR (557196, 1:200, BD Biosciences). Mouse isotype controls were from BD Biosciences and each was used at 1:500. Surface and intracellular molecular staining was carried out as described elsewhere^21^,^49^.

### Immunoprecipitation and immunoblotting

For immunoprecipitation and immunoblotting, cells were extracted in ice-cold Nonidet P-40 (NP-40) extraction buffer (20 mM Tris-HCl, pH 7.5, containing 1 mM EDTA, 150 mM NaCl, 2.5 mM sodium pyrophosphate, 1 mM β-glycerophosphate, 1% NP-40, 1 mM phenylmethyl sulphonyl fluoride and 1 μg per ml aprotinin). Cell extracts were centrifuged at 10,000*g* for 10 min at 4 °C and immunoprecipitated with 1 μg of mouse anti-DR/DP monoclonal antibody (sc-51617, Santa Cruz Biotechnology, Santa Cruz, CA), 1 μg of mouse anti-DP monoclonal antibody (H127, Leinco Technologies Inc., St Louis, MO), 1 μg of mouse anti-DRα monoclonal antibody (sc-53499, Santa Cruz Biotechnology) or 1 μg of mouse anti-CLIP monoclonal antibody (sc-12725, Santa Cruz Biotechnology) on 20 μl of protein G-Sepharose (Santa Cruz Biotechnology) at 4 °C overnight. The beads were isolated by centrifugation and washed four times with ice-cold NP-40 extraction buffer. The bound proteins were separated by Tris-Glycine SDS-PAGE followed by electrophoretic transfer to Immobilon-P membrane (Millipore, Bedford, MA). Small peptides were separated by Tris-Tricine SDS-PAGE. After blocking with 0.1% Tween 20 in Tris-buffered saline, the membranes were incubated with the indicated primary antibodies at 4 °C overnight, washed and incubated with horseradish peroxidase (HRP)-conjugated goat anti-mouse IgG (H+L) secondary antibody (Promega, Madison, WI) or HRP-conjugated rat anti-mouse IgG VeriBlot secondary antibody (Abcam, Cambridge, MA), which is only capable of recognizing native, non-denatured primary mouse antibody, at room temperature for 1 h. The signal was detected by enhanced chemiluminescence (GE Healthcare). Antibodies to the following proteins were used in immunoblots: mouse anti-Ii monoclonal antibodies (sc-6262, 1:1,000, and sc-47742, 1:500, Santa Cruz Biotechnology), mouse anti-DR/DP monoclonal antibody (sc-51617, 1:1,000, Santa Cruz Biotechnology), mouse anti-DR/DP monoclonal antibody (sc-51617, 1:2,000, Santa Cruz Biotechnology), mouse anti-DP monoclonal antibody (H127, 1:1,000, Leinco Technologies), mouse anti-DRα monoclonal antibody (sc-53499, 1:1,000, Santa Cruz Biotechnology), mouse anti-CLIP monoclonal antibody (sc-12725, 1:3,000, Santa Cruz Biotechnology), mouse anti-MAGE-A3 monoclonal antibody (H00004102-M0, 1:1,000, Novus Biologicals) and mouse anti-HLA class I monoclonal antibody (ab70328, 1:2,000, Abcam). All immunoblotting experiments were repeated a minimum of three times. Full, uncropped blots are provided in Supplementary Figs 7 and 8.

### *In vivo* chemical crosslinking

Intracellular crosslinking was performed using DSP. For each experiment, crosslinkers were freshly prepared as a 4 mg ml^−1^ solution in dimethyl sulfoxide and diluted to the indicated final concentrations in PBS. Cells were incubated with DSP on ice for 2 h. After removal of DSP, cells were incubated on ice for 10 min with quenching solution. Quenching solution was then removed and cell lysates were extracted in ice-cold NP-40 extraction buffer.

### Peptide elution assay

A total of 2 × 10^8^ cells of each T2 transfectants and EBV-LCL were harvested, washed twice in PBS, and resuspended in 1% NP-40 extraction buffer. The lysates from T2 tranefctants and EBV-LCL were affinity purified using 2 mg of anti-pan HLA class II monoclonal antibody (I3, in-house) or anti-DP monoclonal antibody (H127, Leinco Technologies) immobilized on an AminoLink Plus column. The HLA-DP-bound peptides were eluted by boiling in 10% acetic acid solution and concentrated by a 10-kDa cutoff membrane (Thermo Scientific, Canada). The peptides were desalted by a Sep-Pak C18 cartridge (Waters, Milford, MA) as described previously^32^. Desalted peptides were analysed using reverse-phase liquid chromatography coupled to a tandem mass spectrometer, as described previously^50^. Briefly, peptides were separated based on hydrophobicity on a 50-cm reverse phase C18, ES803 (Thermo Scientific) nano-flow column. A 140-min gradient delivered using an Easy-1,000 nanoLC system (Thermo Scientific) with a flow rate of 250 nl min^−1^ was utilized for chromatographic separation of peptides. The eluted peptides were detected on a QExactive tandem mass spectrometer (Thermo Scientific) operating in Top10 mode. MS1 spectra were obtained at 70,000 resolution with 120-ms injection time, whereas MS2 spectra were obtained at 17,500 resolution and 100-ms injection time, with an isolation width of 2 *m*/*z* and 25 normalized collision energy. The acquired raw data from the tandem mass spectrometer were searched using MaxQuant (version 1.3.0.3). A UniProt human FASTA file combined with a bovine FASTA file (version: 2012-07-19, number of sequences: 44,107) was used as the source for protein sequences. To search for all possible peptides, enzyme specificity was defined as unspecific, and oxidation of methionine, in addition to N-terminal protein acetylation, were defined as variable modification. Searches were performed with 10 p.p.m. mass error tolerance and false-positive discovery of peptides was controlled using a target-decoy approach with 1% false discovery rate.

### Confocal microscopic analysis

Cells grown on coverslips were fixed with 4% paraformaldehyde in PBS for 15 min at room temperature and permeabilized with 0.1% Triton X-100 in PBS for 20 min at room temperature. For immunostaining, the cells were incubated with various primary antibodies at 37 °C for 1 h, washed three times with PBS and incubated with appropriate secondary antibody, anti-rabbit IgG conjugated with Dylight405 (711-475-152, 1:1,000, Jackson ImmunoResearch, West Grove, PA) or anti-mouse conjugated with Alexa488 (715-545-150, 1:1,000, Jackson ImmunoResearch) at 37 °C for 1 h. The coverslips were washed three times with PBS and incubated with anti HLA-DP monoclonal antibody conjugated with DyLight594 (H1584, 1:200, Leinco Technologies) at 37 °C for 1 h, washed three times with PBS and mounted on a slide with antifade medium (Vectashield, Vector Labs, Burlingame, CA). Fluorescence images were captured using a confocal microscope (Zeiss LSM700). The primary antibodies utilized were as follows: rabbit anti-Ii polyclonal antibody (sc-20082, 1:200, Santa Cruz Biotechnology), mouse anti-Ii monoclonal antibody (sc-6262, 1:200, Santa Cruz Biotechnology), mouse anti-EEA1 monoclonal antibody (610457, 1:250, BD Biosciences), mouse anti-LAMP1 monoclonal antibody (sc-20011, 1:100, Santa Cruz Biotechnology) and mouse anti-PDI monoclonal antibody (MA3-019, 1:500, Thermo Scientific).

### pH-dependent class II dissociation assay

Lysates were immunoprecipitated with anti-DPβ monoclonal antibody crosslinked to agarose beads (Thermo Scientific). The beads were washed three times with 1% NP-40 extraction buffer, followed by incubation with desalting buffer of pH 4.0, 5.0, 6.0, 7.0 or 8.0 for 10 min. The samples were then analysed by immunoblotting.

### *In vitro* T-cell assays

Human T cells were positively purified using magnetic beads (Miltenyi Biotec) and transduced with either the DP4/MAGE-A3_243–258_ TCR genes^45^ or A2/MART1_27–35_ TCR (DMF5) genes^51^. IL-2 and interferon (IFN)-γ ELISPOT, as well as standard chromium release assays, were performed as described previously^49^,^52^,^53^. For the IL-2 ELISPOT assay, PVDF plates (Millipore) were coated with capture monoclonal antibody (SEL002; R&D Systems, Minneapolis, MN). T cells were incubated with 2 × 10^4^ stimulator cells for 20–24 h at 37 °C. Plates were washed and incubated with biotin-conjugated detection monoclonal antibody (SEL002; R&D Systems). After washing, alkaline phosphatase-conjugated streptavidin (Jackson ImmunoResearch) was added. Plates were washed and incubated with nitroblue tetrazolium/5-bromo-4-chloro-3-indolyl phosphate (Promega) and IL-2 spots were developed. For the IFN-γ ELISPOT assay, polyvinylidene difluoride (PVDF) plates (Millipore) were coated with capture monoclonal antibody (1D1K; MABTECH, Mariemont, OH). T cells were incubated with 2 × 10^4^ stimulator cells for 20–24 h at 37 °C. Plates were washed and incubated with biotin-conjugated detection monoclonal antibody (7-B6-1; MABTECH). HRP-conjugated SA (DAKO, Carpenteria, CA) was then added, and IFN-γ spots were developed. For the standard chromium release assay, 5 × 10^3^ target cells that were labelled with ^51^chromium were mixed with effector cells for 4 h at 37 °C in a 96-well V-bottom plate. Percent-specific lysis was calculated ((experimental result−spontaneous release)/(maximum release−spontaneous release)) × 100%. The peptides used were MAGE-A3_243–258_ (KKLLTQHFVQENYLEY; Genway, San Diego, CA), tetanus toxin_947–967_ (FNNFTVSFWLRVPKVSASHLE; Genway), MART1_27–35_ (AAGIGILTV; ProImmune, Oxford, UK) and HIV pol_476–484_ (ILKEPVHGV; ProImmune).

### *In vivo* T-cell assays

NSG mice (*n*=3 per group) were subcutaneously inoculated with 2 × 10^6^ K562 cells stably expressing Ii and native MAGE-A3 in conjunction with DP4 (DP4/Ii/MAGE-A3) or DP4^84DEAV87^ (DP4^84DEAV87^/Ii/MAGE-A3). Two days later, the mice were infused with 4 × 10^7^ untransduced or DP4/MAGE-A3_243–258_ TCR-transduced CD3^+^ T cells. Tumour volume was calculated using the formula: tumour volume (mm^3^)=length × width × height × 0.52. Data are shown as means±s.e.m.'s for each group (*n*=3). There was no significant difference in the tumorigenicity of the two cell lines. Mice were monitored, at minimum, once every 3 days and tumours were not to exceed 1.5 cm in diameter, nor 1,500 mm^3^ in volume.

### Statistical analysis

Statistical analysis was performed using GraphPad Prism 5.0. Unpaired, two-tailed Welch's *t*-test was used for two-sample comparisons. *P* values of <0.05 were considered significant. All analyses were performed using the GraphPad Prism software. No statistical method was used to predetermine sample size. The investigators were not blinded to allocation during experiments and outcome assessment. The experiments were not randomized.

### Data availability

Mass spectrometry data that support the findings of this study have been deposited in the Mass Spectrometry Interactive Virtual Environment with the primary accession code MSV000080228. The authors declare that all other data that support the findings of this study are available within the article and its Supplementary Information files, or are available from the authors upon request.

## Additional information

**How to cite this article:** Yamashita, Y. *et al*. HLA-DP^84Gly^ constitutively presents endogenous peptides generated by the class I antigen processing pathway. *Nat. Commun.* **8**, 15244 doi: 10.1038/ncomms15244 (2017).

**Publisher's note:** Springer Nature remains neutral with regard to jurisdictional claims in published maps and institutional affiliations.

## Supplementary Material

Supplementary Information Supplementary Figures and Supplementary Tables.

## Figures and Tables

**Table 1:**
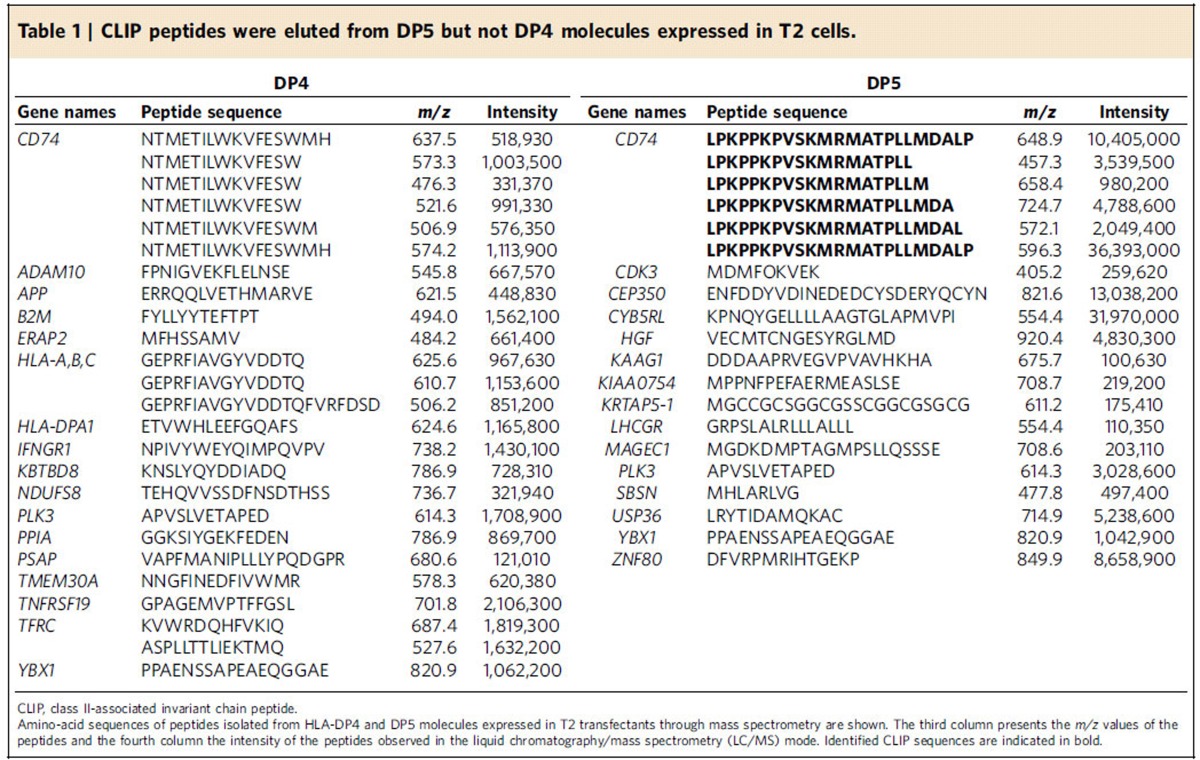
CLIP peptides were eluted from DP5 but not DP4 molecules expressed in T2 cells.
