# Supplementary material for: HLA-DP84Gly constitutively presents endogenous peptides generated by the class I antigen processing pathway
Source: Nat Commun. 2017 May 10;8:15244. doi: 10.1038/ncomms15244 (PMC5436232; doi:10.1038/ncomms15244)
Supplement: Supplementary Information — Supplementary Figures and Supplementary Tables. [file ncomms15244-s1.pdf]

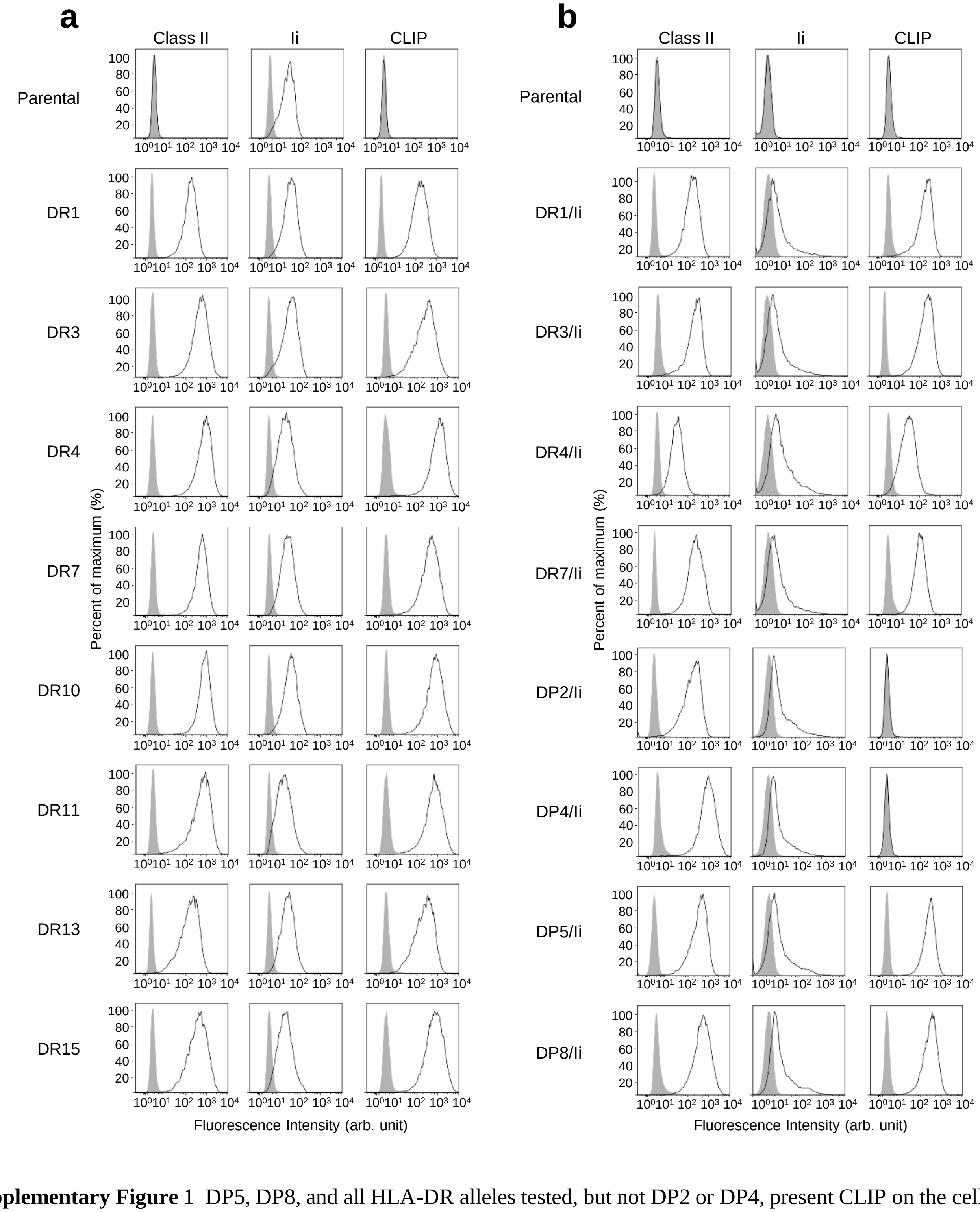

Supplementary Figure 1 DP5, DP8, and all HLA-DR alleles tested, but not DP2 or DP4, present CLIP on the cell surface. (**a**) T2 cells were stably transduced with *DRA1*0101* (DRA1) along with *DRB1*0101* (DRB1), *DRB1*0103* (DRB3), *DRB1*0401* (DRB4), *DRB1*0701* (DRB7), *DRB1*1001* (DRB10), *DRB1*1101* (DRB11), *DRB1*1301* (DRB13) or *DRB1*1501* (DRB15). Surface expression of class II, Ii, and CLIP on these T2 transfectants and parental T2 cells was analyzed by flow cytometry following staining with specific mAbs. (**b**) K562 cells were stably transduced with both Ii and DRA1 along with DRB1, DRB3, DRB4 or DRB7. K562 cells were stably infected with both Ii and DPA1 in conjunction with DPB2 (DP2), DPB4 (DP4), DPB5 (DP5) or DPB8 (DP8). Surface expression of class II, Ii, and CLIP on the K562 transfectants and parental K562 were analyzed by flow cytometry after staining with specific mAbs.

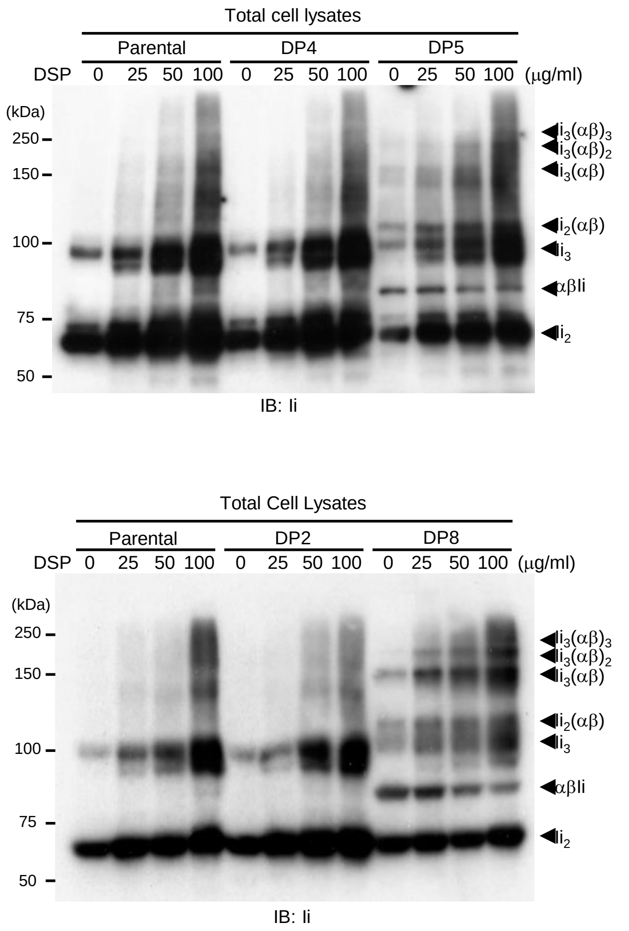

Supplementary Figure 2 DP^{84DEAV87} but not DP^{84GGPM87} forms multimers with endogenous Ii. T2 transfectants stably expressing DP^{84DEAV87} (DP5 or DP8) or DP^{84GGPM87} (DP2 and DP4) and parental T2 were treated by DSP at the indicated concentrations for 2 hrs. Non-reduced samples were immunoblotted with anti-Ii mAb**.**

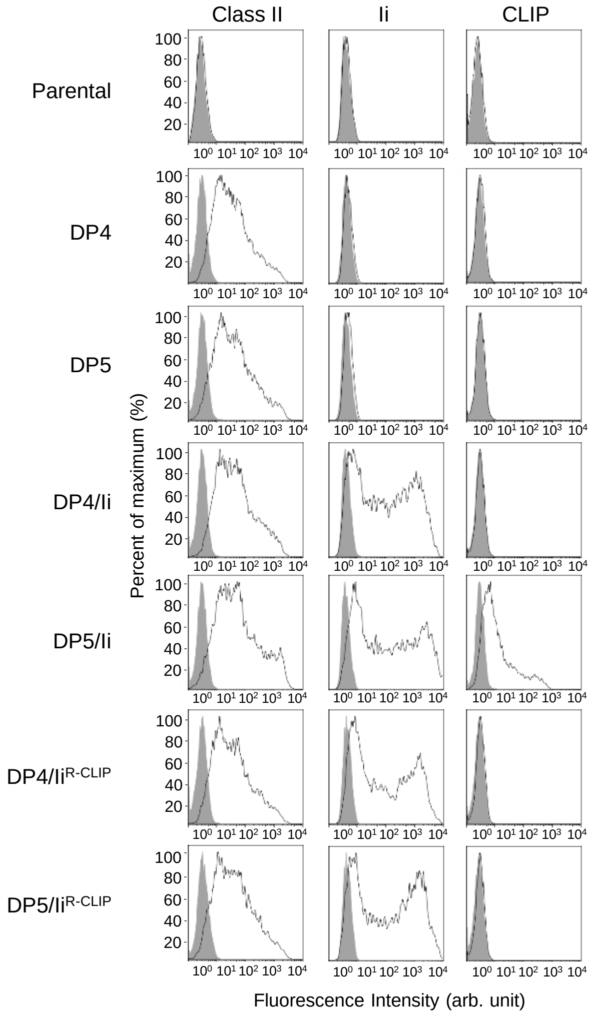

Supplementary Figure 3

Surface expression of Class II, Ii, and CLIP on HEK293 transiently transfected with *DPA1*01:03* in conjunction with either *DPB1*04:01* (DP4) or *DPB1*05:01* (DP5) and one of *Ii* or *Ii*$^{R\text{-}CLIP}$ as indicated. Expression of Class II, Ii, and CLIP on HEK293 transfectants and parental HEK293 were analyzed by flow cytometry following staining with specific mAbs.

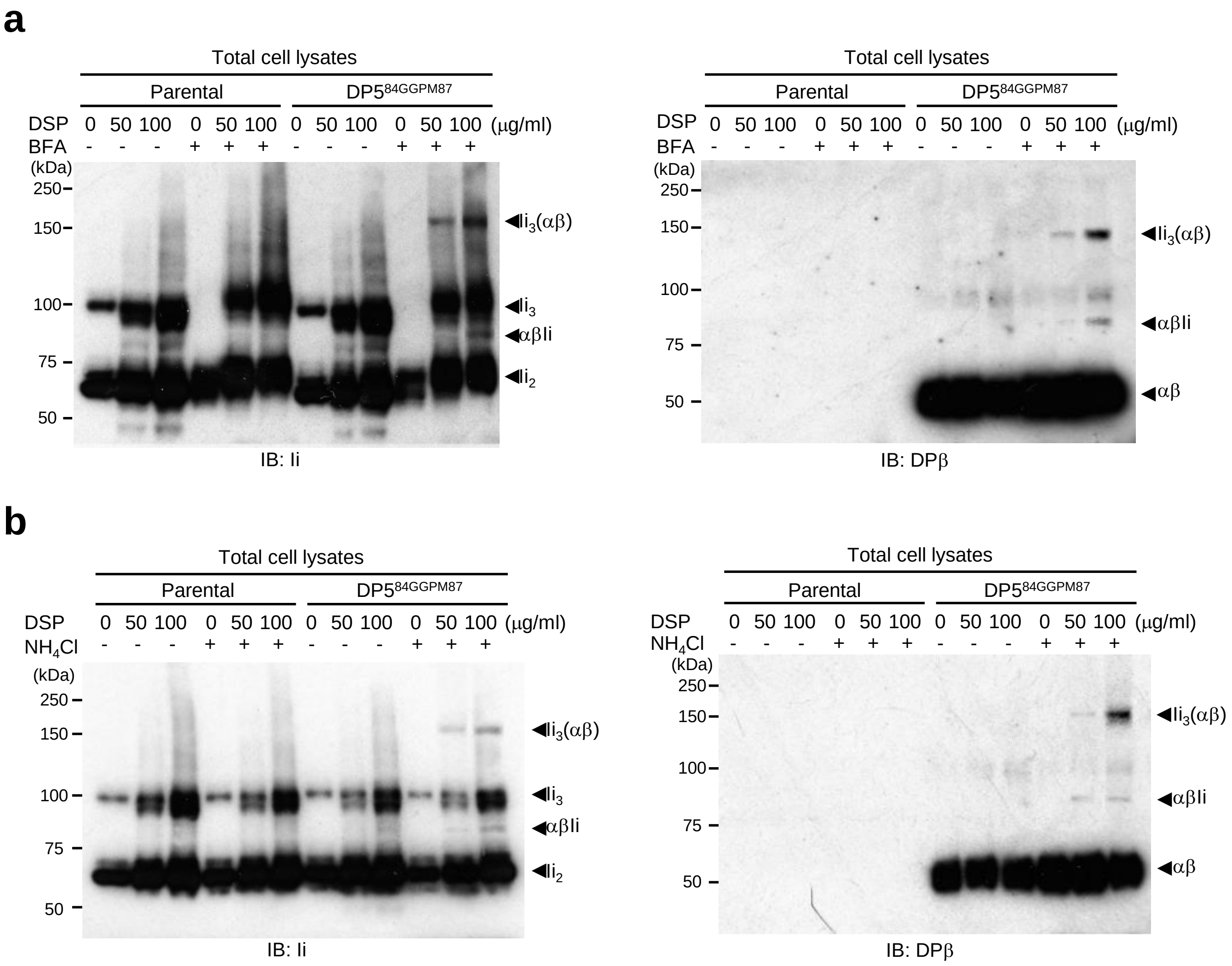

Supplementary Figure 4 DP5^{84GGPM87} associates with Ii in neutral pH conditions. (**a**,**b**) T2 and mutant T2/DP5^{84GGPM87} cells were cultured in the presence or absence of 10 mg/ml BFA (**a**) or 40 mM ammonium chloride (NH_4Cl) (**b**). The cells were further treated by DSP at the indicated concentrations for 2 hrs. Non-reduced samples were immunoblotted with anti-Ii or DPβ mAb.

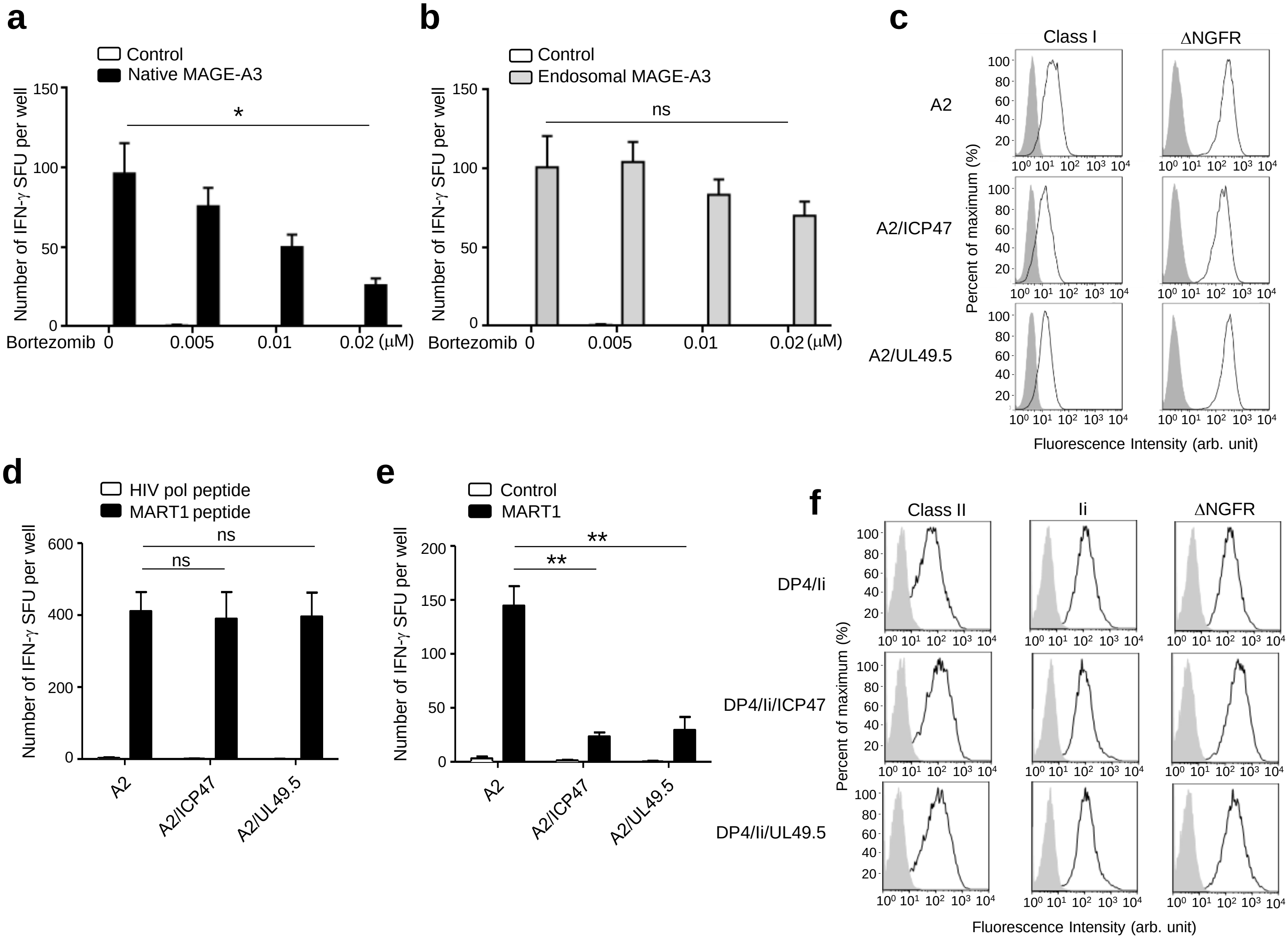

Supplementary Figure 5 Role of the proteasome and TAP molecules in the processing and presentation of DP4-restricted peptides. (**a,b**) K562/DP4/Ii cells were transiently transfected with a retrovirus vector encoding IRES-EGFP (control) or a native (**a**) or endosome-targeted (**b**) form of MAGE-A3 linked with IRES-EGFP. The cells were cultured with bortezomib at the indicated concentrations for 48 hrs. Transient transfection efficiencies were normalized to EGFP expression measured by flow cytometry. Data shown represent means ± SDs of triplicates. (**c**) K562/A2 were stably transfected with a retrovirus vector encoding IRES-ΔNGFR (control), or ICP47 or UL49.5 linked with IRES-ΔNGFR. ΔNGFR transduced cells were isolated using anti-NGFR mAb. Surface expression of class I and ΔNGFR (ICP47 or UL49.5) was analyzed by flow cytometry after staining with specific mAbs. (**d**) A2/MART1$_{27\text{-}35}$ T cells were stimulated with the K562-based aAPCs pulsed with A2-restricted HIV pol$_{476\text{-}484}$ (control) or MART1$_{27\text{-}35}$ peptide, and IFN-γ secretion was evaluated by ELISPOT assays. Data shown represent means ± SDs of triplicates. (**e**) The indicated K562 aAPCs were transiently transfected with a retrovirus vector encoding IRES-EGFP (control) or full-length MART1 gene linked with IRES-EGFP. Transient transfection efficiencies were normalized to EGFP expression measured by flow cytometry. A2/MART1$_{27\text{-}35}$ T cells were stimulated with the indicated aAPCs and IFN-γ secretion was measured by ELISPOT analysis. Data shown represent means ± SDs of triplicates. Results are representative of three independent experiments. ns, not significant; *p<0.05, **p<0.01 by unpaired, two-tailed Welch's *t* test. (**f**) K562/DP4/Ii cells were stably transfected with a retrovirus vector encoding IRES-ΔNGFR (control), or ICP47 or UL49.5 linked with IRES-ΔNGFR. ΔNGFR transduced cells were isolated using anti-NGFR mAb. Surface expression of class II and ΔNGFR (ICP47 or UL49.5), as well as intracellular expression of Ii, were analyzed by flow cytometry after staining with specific mAbs.

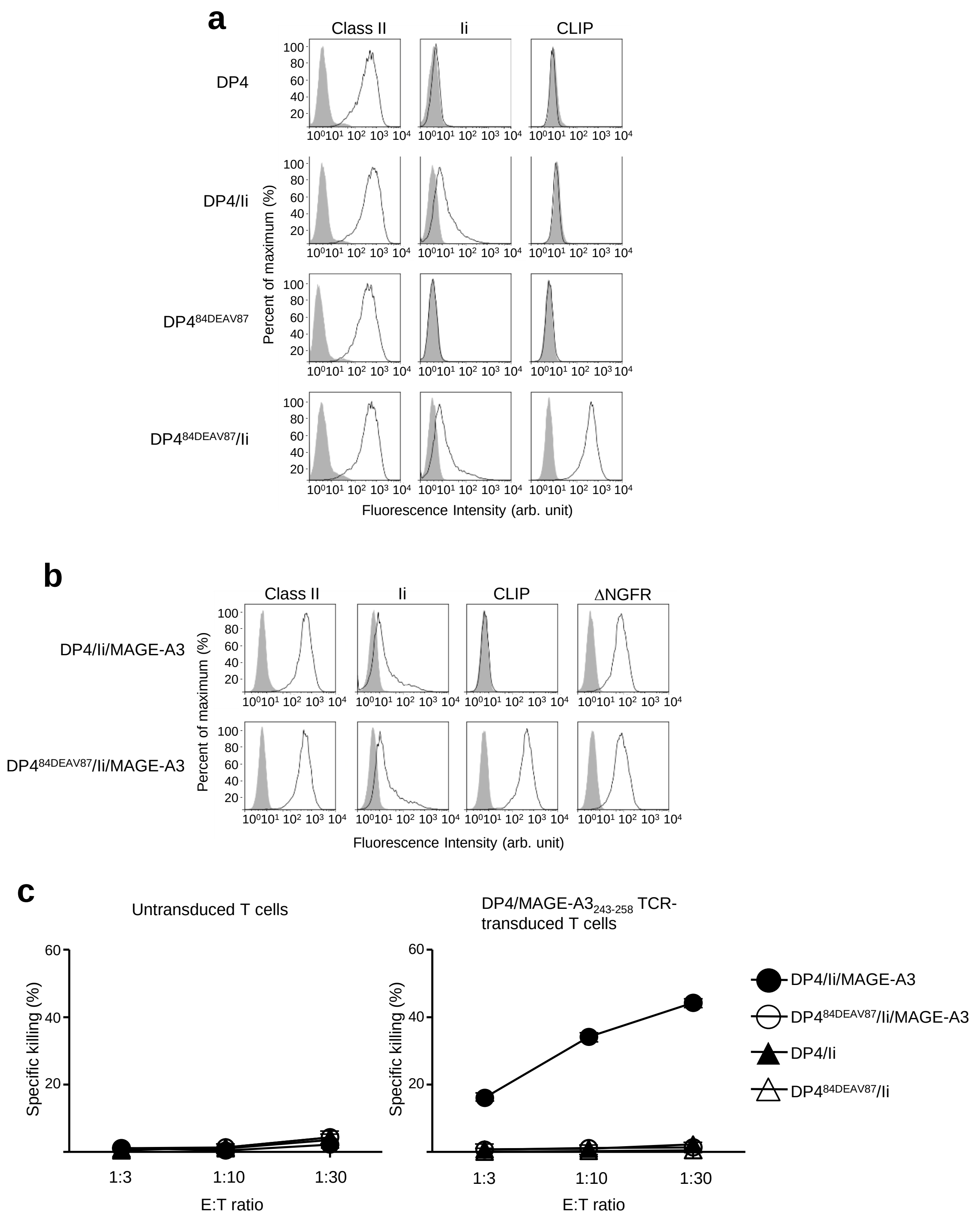

Supplementary Figure 6 $DP^{84GGPM87}$ constitutively presents endogenous peptides even in the presence of Ii and can be targeted by antigen-specific T cells. (**a**) K562 cells expressing DP4 or $DP4^{84DEAV87}$ were stably transduced with Ii. Surface expression of class II, Ii, and CLIP were studied by flow cytometry following staining with specific mAbs. (**b**) A native form of MAGE-A3 was fused with ΔNGFR as a cell-surface marker for tracking and sorting of transduced cells. ΔNGFR (MAGE-A3)-transduced cells were isolated using anti-NGFR mAb. Surface expression of class II, Ii, CLIP, and ΔNGFR (MAGE-A3) is depicted. (**c**) $CD8^+$ T cells transduced with or without DP4/MAGE-$A3_{243\text{-}258}$ TCR were coincubated with K562 cells stably expressing the indicated genes at various effector/target ratios. Cytotoxicity was assessed by a standard ^{51}Cr release assay. The results are presented as the means ± SDs of triplicates.

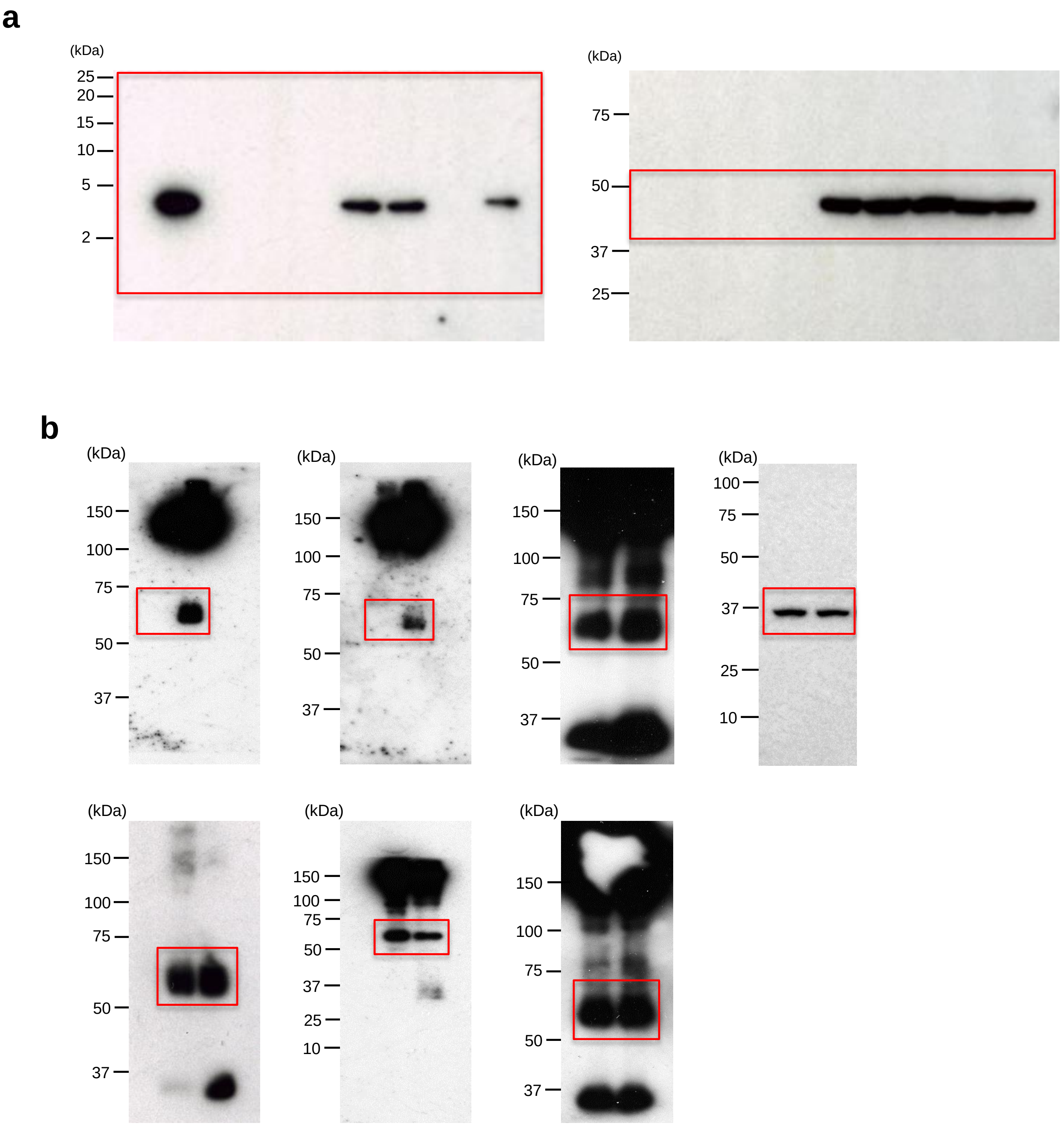

Supplementary Figure 7: Uncropped western blotting images from Figures 2a (**a**) and 2c (**b**). Outlined regions represent portion of each image displayed in main figures.

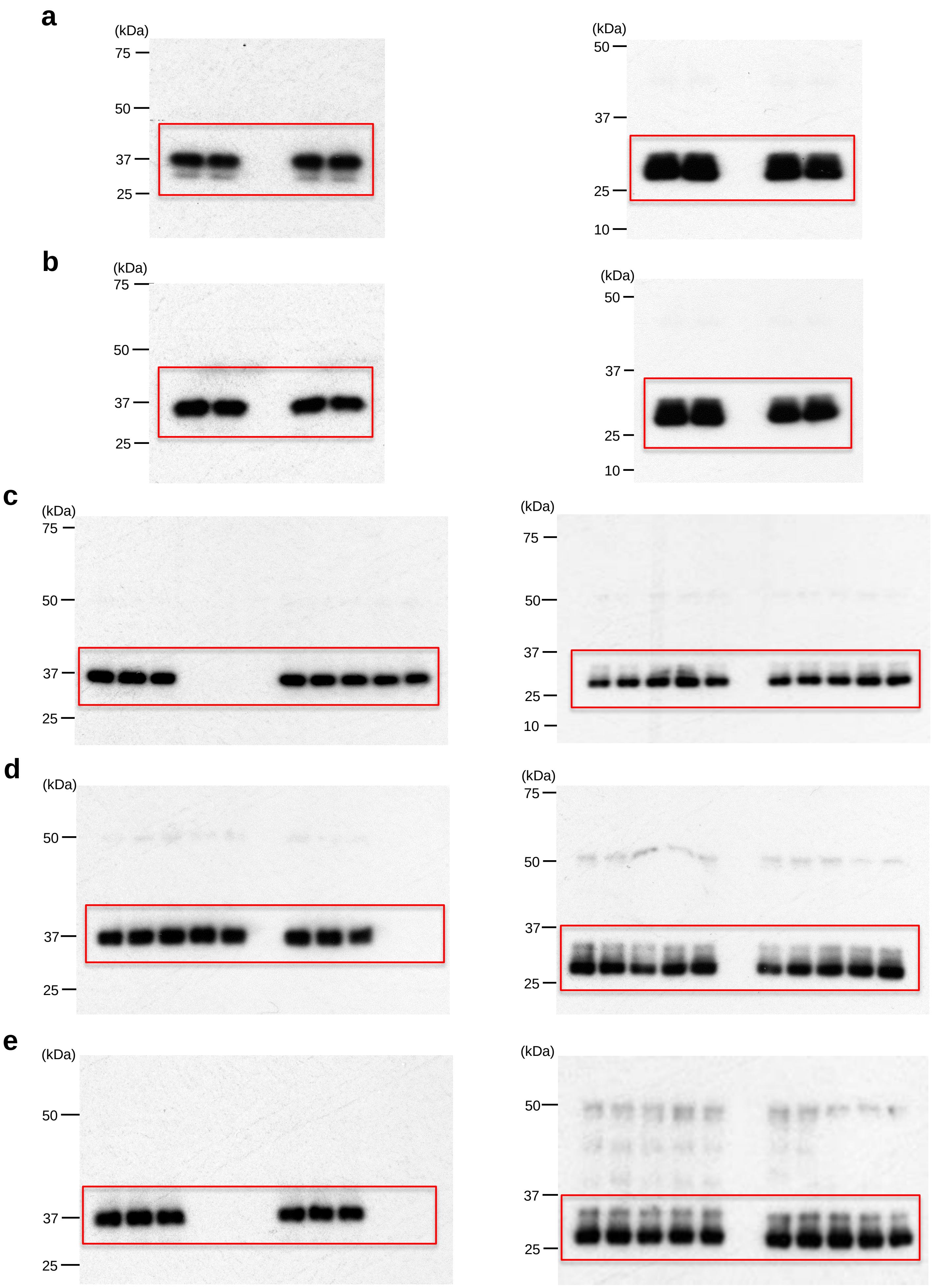

Supplementary Figure 8: Uncropped western blotting images from Figure 5a **(a)**, 5b **(b)**, 5c (**c**), 5d (**d**), and 5e **(e).** Outlined regions represent portion of each image displayed in main figures.

DP4/4 EBV-LCL			
Gene names	Peptide sequence	m/z	Intensity
ABHD3	SSVNKHRHMFVKQVDM	647.3	409,650
ARIH2	MSVDMNSQGSDSNEEDY	635.6	248,600
ATP13A2	SVLALLGTIYSIFIL	405.5	3,023,500
ATP5B	PLDSTSRIMD	566.8	3,045,000
B2M	TPKIQVYSRHPAEN	546.3	1,975,500
BRP44L	AGALVRKAAD	485.3	2,573,200
BST2	AQGQKKVEELEGEIT	828.9	305,170
CACNA1D	MMMMMMMKKMQHQRQ	666.6	556,700
CCRL1	MALEQNQSTDYYYEE	941.4	786,110
CLMP	MSLLLLLLLVSY	458.9	2,015,800
CMTM6	MENGAVYSPTTEED	770.8	1,183,100
COX5A	SHGSQETDEEFD	689.8	118,320
COX5B	PYNVLAPKGASGTRED	557.9	7,623,200
CTSH	LPSQAFEYILYNKG	547.3	1,193,000
DCAF15	MLMNMMMMSDENHRD	628.2	221,030
EPRS	ATLSLTVNSGD	538.3	2,402,700
ERV3-1	DQWPWEARELMP	518.9	163,690
FRRS1L	MRRPRQGGG	506.8	1,548,300
GEMIN2	RRAELAGLK	506.3	251,100
HLA-A,B,C	EPRFISVGYVDDTQ	812.4	1,460,900
HLA-DPB1	SPVTVEWKAQ	571.8	14,912,000
	SPVTVEWKAQSD	672.8	2,695,800
IFITM1,2,3	VGDVTGAQAYASTAK	718.9	200,210
IGLL1	TPSKQSNNKYAASSYLSLTP	718.7	2,549,300
	SNNKYAASSYLSLTPE	871.9	11,970,000
	NNKYAASSYLSLTPE	828.4	8,742,100
	NNKYAASSYLSLTP	763.9	3,323,900
	SNNKYAASSYLSLTP	807.4	3,290,900
	NNKYAASSYLSLT	715.4	2,271,400
	NKYAASSYLSLTPE	771.4	1,613,100
KARS	AAVQAAEVKVD	549.8	1,389,600
MT1B	MDPNCSCTTGGSCACA	506.5	3,192,300
NDUFB11	PSKIQLPEDE	577.3	4,704,200
NDUFS8	TYKYVNMQD	580.3	835,150
NDUFV2	GAGGALFVHRD	549.3	2,894,700
NMNAT3	MKSRIPVVLL	384.9	556,190
NPIPL1,2	MRLRFWLLIWLLL	590.7	294,990
OR2T1	LSLIDMMYISTI	699.4	2,271,400
PCDH19	ESLLLPVLLLLAI	703.0	961,180
PGAM1,4	VPQIKEGKRVLIAAHG	571.7	1,015,600
PKM2	IVLTKSGRSAHQVARY	595.0	3,119,200
PPIA	GGKSIYGEKFEDEN	523.9	35,131,000
	GKSIYGEKFEDEN	757.3	4,778,600
	GGKSIYGEKFEDE	728.8	2,774,400
RPS12	AEEGIAAGGVMD	559.2	4,116,300
RPS3	GLMIHSGD	414.2	4,026,400
SLC16A3	GGAVVDEGPTGVKAPD	733.9	672,040
SLC25A13	AAAKVALTKRAD	404.6	8,729,200
SLC25A5	TDAAVSFAKD	511.7	3,861,900
SSR1	MRLLPRLLLLLLL	788.0	584,300
TFRC	GGYVAYSKAATVTGK	735.9	4,492,100
TOMM22	AAAVAAAGAGEPQSPD	690.8	1,203,200
TUBA1A,1B	SVEGEGEEEGEEY	720.8	2,237,200
UQCRH	PEEEEEEEEELVD	801.8	21,037,000
	GLEDEQKMLTESGD	775.3	9,333,800
VAPA	ASASGAMAKHEQILVLD	869.9	643,880
YBX1	PPAENSSAPEAEQGGAE	819.8	12,425,000
ZUFSP	PFCGKIEEHSEDMETH	943.9	309,150

DP5/17 EBV-LCL			
Gene names	Peptide sequence	m/z	Intensity
ACAT1	DAAKRLNVTPLARIV	409.0	264,330
ACTB	DDDIAALVVD	522.2	4,124,800
APOB	LKKTKNSEEFAAAMS	826.9	10,240,000
	LKKTKNSEEFAAAM	783.4	5,346,000
ATP5B	PLDSTSRIMD	566.8	2,003,400
ATP5H	AGRKLALKTID	394.9	1,554,200
ATP5J	PKFEVIEKPQA	642.4	3,758,000
BRP44L	AGALVRKAAD	485.3	1,253,100
BST2	VERLRRENQVLSVR	438.3	6,134,100
	AQGQKKVEELEGE	721.9	2,483,400
CD74	LDKLTVTSQNLQ	679.4	11,526,000
	GRLDKLTVTSQNLQ	785.9	10,530,000
	PSSGLGVTKQDLGPVPM	840.9	1,899,600
	**SKMRMATPLLMQ**	702.9	1,968,300
COX5B	PYNVLAPKGASGTRED	557.9	3,672,200
ENTPD2	MAGKVRSLLPPLLLAAA	430.0	10,653,000
EPRS	ATLSLTVNSGD	538.3	3,070,300
FARSA	AEPRPPPTQEAA	631.3	1,542,100
FCER2	KGTKQWVHARYA	481.3	15,359,000
	GKGTKQWVHARYA	500.3	8,773,200
GALNT2	TPEQRRSRQGNPVAPI	601.7	8,845,800
GAPDH	EHQVVSSDFNSDTHS	562.6	5,033,600
HLA-A	RFLRGYHQYAYDGKD	472.0	10,893,000
HLA-A,B,C	EPRFIAVGYVDDTQ	804.4	935,560
HLA-B	GRLLRGHNQYAYDGKD	620.6	27,976,000
	GRLLRGHNQYAYDGKDY	506.2	8,254,300
	GRLLRGHNQYAYDGK	436.7	2,972,500
	RLLRGHNQYAYDGKDY	492.0	1,532,100
	LLRGHNQYAYDGKD	549.6	1,814,800
	YDGKDYIALNEDLSS	850.9	5,705,000
	YDGKDYIALNEDLS	807.4	2,407,600
	GKDYIALNEDLSS	711.8	1,117,500
HLA-DPA1	PPEVTVFPKE	570.8	2,703,400
HLA-DPB1	SPVTVEWKA	507.8	6,595,100
	SPVTVEWKAQ	571.8	5,533,100
	SPVTVEWKAQSD	672.8	1,591,800
	SPVTVEWKAQSDS	716.3	867,170
HLA-G	YDGKDYLALNED	707.3	2,625,100
	DGKDYLALNEDLR	506.9	2,031,000
HMGCR	MIEVNINKNLVGSAM	543.9	5,138,800
HNRNPC	ASNVTNKTD	474.2	1,531,200
HSPD1	GFEKISKGANPVE	687.4	2,965,600
IFNGR1	TVIKAPTSFGYDKP	507.6	2,098,300
IGHM	RGGKYAATSQVLLPS	515.6	16,501,000
	RGGKYAATSQVLLPSKD	596.7	3,620,000
KARS	AAVQAAEVKVD	549.8	937,560
KCNH3,4	MHAVVFGNVTAIIQ	749.4	1,486,500
LAPTM5	NVRIATTALAIYH	480.6	1,226,300
LRP1	LTPPLLLLL	495.8	833,660
MAST1	LLISSLLQTNPLVRLG	434.0	476,290
MX1	VVSEVDIAKAD	572.3	5,701,600
MYH10	AQRTGLED	444.2	1,491,600
MYH9	EEVDGKADGAEAKPAE	538.2	75,577,000
	GSDEEVDGKADGAEAKPAE	624.6	11,079,000
	AQQAADKYLYVD	691.8	4,143,100
NCL	TPAKKTVTPAKAVTTPG	555.7	1,912,900
NDUFB11	PSKIQLPEDE	577.3	3,471,300
NDUFV2	GAGGALFVHRD	549.3	2,385,400
OTOF	LRAQVKRHTVRDKLR	468.8	1,454,300
PBX1,2	MRLDNMLLAEGVAGP	792.9	4,003,400
PGAM1,4	VPQIKEGKRVLIAAHGN	609.7	6,500,800
PHB2	ESFTRGSDSLIKGKK	550.6	3,225,800
PKM2	IVLTKSGRSAHQVAR	540.6	11,473,000
	IVLTKSGRSAHQVARYRPR	548.6	9,241,800
PPIA	GGKSIYGEKFEDEN	523.9	17,408,000
RPL30	PGDSDIIRSMPEQTGEK	619.6	2,426,800
RPS19	LDRIAGQVAAANKKH	530.3	2,139,400
RPS3	AVQISKKRKFVAD	496.3	20,504,000
RPS5	TEWETAAPAVAETPD	793.4	727,340
RTF1	GRAAAAAAAVAVPLA	426.2	599,320
S100A10	PSQMEHAMETMMF	784.3	2,049,500
SCN7A	PSLVQLILLSRIIHML	922.6	2,452,100
SEMA7A	GEMRGYAPFSPDENS	827.8	984,840
	EMRGYAPFSPDEN	755.8	652,350
SERPINA5	MQLFLLLCLVLLSP	801.0	1,033,400
SLC25A13	AAAKVALTKRAD	404.6	1,053,900
SLC25A5	TDAAVSFAKD	511.7	1,323,700
SLC9A8	SPVTQILMQ	507.8	4,575,300
TFRC	NPGGYVAYSKAATVTG	777.4	6,442,500
	NPGGYVAYSKAATVTGK	561.0	3,412,400
	GGYVAYSKAATVTG	671.8	3,309,000
	NPGGYVAYSKAATVT	748.9	1,170,300
TGOLN2	DSPSKSGSEAQTTKDVPN	615.6	2,650,300
TOMM22	AAAVAAAGAGEPQSPD	690.8	862,960
TUBA1A,1B	SVEGEGEEEGEEY	720.8	3,664,700
TUBB	ATAEEEEDFGEEAEEEA	941.9	2,416,500
UQCRH	GLEDEQKMLTESGD	775.3	5,436,300
VAPA	ASASGAMAKHEQILVLD	869.9	1,950,400
YBX1	PPAENSSAPEAEQGGAE	819.8	23,961,000

Supplementary Table 1 Repertoires of peptides eluted from endogenously expressing DP4/4 and DP5/17 EBV-LCL. Gene names and amino acid sequences of peptides isolated from DP4/4, homozygous for $DP^{84GGPM87}$, or DP5/17, homozygous for $DP^{84DEAV87}$, EBV-LCL are shown in the first and second columns, respectively. The third column presents the m/z values of the peptides, and the fourth column the intensity of the peptides observed in LC/MS mode. Identified CLIP sequences are indicated in bold.

Primates	DP^{84Gly} allele	DP^{84Asp} allele
Crab-eating macaques (*M. fascicularis*)	–	+
Rhesus macaques *(M. mulatta*)	–	+
Bornean orangutans (*P. pygmaeus*)	–	+
Western gorillas (*G. gorilla*)	–	+
Bonobos (*P. paniscus*)	–	+
Common chimpanzees (*P. troglodytes*)	+	+
Neanderthals (*H. neanderthalensis*)	+	+
Modern Humans (*H. sapiens*)	+	+

Supplementary Table 2 Search results for the presence or absence of DP^{84Gly} and DP^{84Asp} alleles in the genomes of crab-eating macaques (*M. fascicularis*), rhesus macaques (*M. mulatta*), bornean orangutans (*P. pygmaeus*), western gorillas (*G. gorilla*), bonobos (*P. paniscus*), common chimpanzees (*P. troglodytes*), neanderthals (*H. neanderthalensis*) and modern humans (*H. sapiens*).
